# Multiconfigurational actinide nitrides assisted by double Möbius aromaticity†

**DOI:** 10.1039/d4sc01549e

**Published:** 2024-04-26

**Authors:** Xuhui Lin, Xiaoli Lu, Shenghui Tang, Wei Wu, Yirong Mo

**Affiliations:** a School of Physics, Central South University Changsha Hunan 410083 China xuhui.lin@csu.edu.cn; b School of Chemistry, Southwest Jiaotong University Chengdu Sichuan 610031 China; c The State Key Laboratory of Physical Chemistry of Solid Surfaces, iChEM, Fujian Provincial Key Laboratory of Theoretical and Computational Chemistry and College of Chemistry and Chemical Engineering, Xiamen University Xiamen Fujian 361005 China weiwu@xmu.edu.cn; d Department of Nanoscience, Joint School of Nanoscience and Nanoengineering, University of North Carolina at Greensboro Greensboro NC 27401 USA y_mo3@uncg.edu

## Abstract

Understanding the bonding nature between actinides and main-group elements remains a key challenge in actinide chemistry due to the involvement of f orbitals. Herein, we propose a unique “aromaticity-assisted multiconfiguration” (AAM) model to elucidate the bonding nature in actinide nitrides (An_2_N_2_, An = Ac, Th, Pa, U). Each planar four-membered An_2_N_2_ with equivalent An–N bonds possesses four delocalized π electrons and four delocalized σ electrons, forming a new family of double Möbius aromaticity that contributes to the molecular stability. The unprecedented aromaticity further supports actinide nitrides to exhibit multiconfigurational characters, where the unpaired electrons (2, 4 or 6 in naked Th_2_N_2_, Pa_2_N_2_ or U_2_N_2_, respectively) either are spin-free and localized on metal centres or form metal–ligand bonds. High-level multiconfigurational computations confirm an open-shell singlet ground state for actinide nitrides, with small energy gaps to high spin states. This is consistent with the antiferromagnetic nature observed experimentally in uranium nitrides. The novel AAM bonding model can be authenticated in both experimentally identified compounds containing a U_2_N_2_ motif and other theoretically modelled An_2_N_2_ clusters and is thus expected to be a general chemical bonding pattern between actinides and main-group elements.

## Abstract 2

Actinide nitride An_2_N_2_ (An = U, Pa, Th and Ac) clusters were characterized by a new family of double Möbius aromaticity, which enables unpaired electrons spin-freely to localize on actinides or form chemical bonds with ligands.

## Graphical abstract

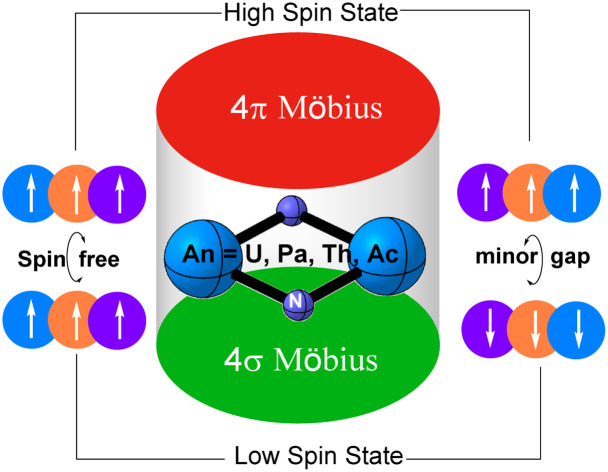

## Introduction

Actinide chemistry, despite its fundamental and practical significance, remains one of the most unexplored domains in the periodic table.^1–4^ Notably, uranium–nitrogen chemistry is of vital importance as uranium nitrides have the potential to be next-generation nuclear fuels.^5^ Furthermore, most recently there has been growing interest in uranium nitrides due to their high reactivity towards N_2_ fixation and small-molecule activation.^6–10^ For instance, uranium nitride materials have been used as catalysts in the Haber–Bosch process for the synthesis of NH_3_ from N_2_ and H_2_.^11–13^ Beyond practical applications in nuclear energy and catalytic processes, interest in uranium nitrides also arises from their fundamental role in understanding the chemical bonding of molecular actinides where f orbitals are involved.^14–16^ Although so far only three uranium nitride solids have been well-identified,^17^ a plethora of molecular species containing terminal and bridged uranium nitrides have been reported over the past few decades.^18–22^ But the abundance of molecular uranium nitrides has not been translated into a comprehensive understanding of the uranium–nitrogen bonding nature, which lags far behind the well-established bonding regimes between uranium and other main-group elements.^23–25^ Accordingly, the development of rational synthetic routes to molecular uranium nitrides and their applications is still at an early stage.^26^

**Fig. 1:**
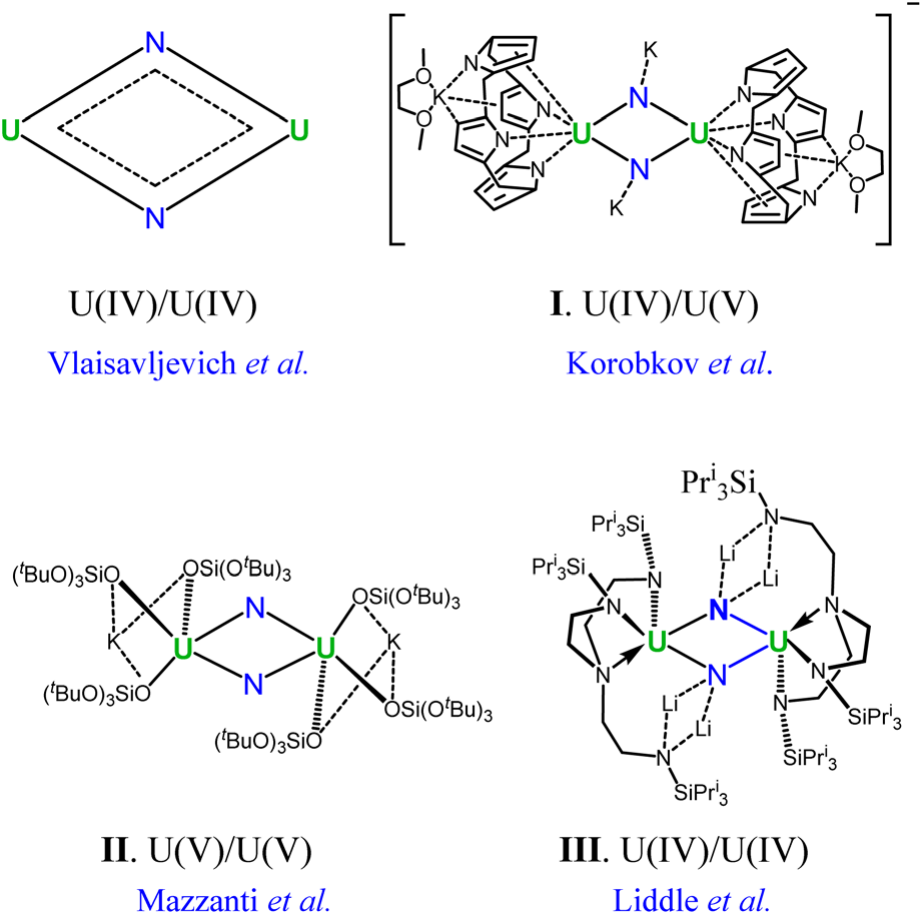
Experimentally identified compounds featuring the U_2_N_2_ nitride motif.

In this context, the U_2_N_2_ nitrides emerge as an attractive target for gaining an in-depth understanding of the uranium–nitrogen bonding. In 2002, Korobkov and coworkers prepared an anionic compound containing a U(iv)/U(v) motif (I in Fig. 1).^27^ Furthermore, U_2_N_2_ nitrides featuring U(v) and U(iv) centers were subsequently identified by Mazzanti's and Liddle's groups,^28,29^ respectively, and are found to exhibit antiferromagnetic coupling (II and III in Fig. 1). It is worth noting that the crystal structures of I–III are centrosymmetric with the *C*_i_ symmetry, having opposite pairs of U–N ring bonds. Remarkably, Vlaisavljevich *et al.* isolated and recognized the naked U_2_N_2_ cluster with argon matrix-isolated IR absorption spectra.^30^ Despite variations in the oxidized states of uranium in complexes I–III and naked U_2_N_2_, all species exhibit very similar structural geometries of equivalent U–N bonds and electronic structures. This highlights an intrinsic bonding pattern in the four-membered U_2_N_2_ ring.

The chemical bonding to actinide is usually predicted to be local,^31–33^ but the diffusion of the 5f and 6d orbitals enables early actinides to engage in delocalized bonds.^34–37^ Given that aromaticity can provide extra stability and diverse reactivity to conjugated molecules, clusters and materials,^38,39^ enormous efforts have been devoted to identify stable and functional actinide compounds with aromaticity. Nevertheless, such compounds are rather limited so far due to the great challenges in their synthesis and characterization. Liddle *et.al.* prepared a crystalline tri-thorium cluster featuring the first delocalized 3c–2e thorium–thorium bond,^40^ which has been interpreted to have a novel core–shell syngenetic σ-aromaticity.^41^ Most recently, we reported the unprecedented planar double Craig–Möbius aromaticity in diboron protactinium (Pa_2_B_2_),^42^ providing an opportunity for establishing aromatic systems between actinides and main-group elements. Since 5f elements exhibit a strong relativistic effect and multiconfigurational character, experimental tools have generally failed to yield quantitative measurements for f-orbital occupancy with a few exceptions.^43^ Alternatively, modern computational methods provide a complemental approach to study molecular actinide science.^44–46^ In particular, *ab initio* valence bond (VB) methods have the capability to perform either single or multi-reference calculations,^47–50^ and thus are promising to unravel the nature of the chemical bonding in molecular actinides.

In this study, we characterized actinide nitrides, including experimentally identified compounds containing a U_2_N_2_ motif and theoretically modelled An_2_N_2_ (An = Pa, Th, Ac) clusters, as a new family of double Möbius aromatic compounds. Different from the exemplary Pa_2_B_2_,^42^ the double Möbius aromaticity in An_2_N_2_ assists the unpaired electrons on metal centers to exhibit multiconfigurational characters. This bonding model is described as an “aromaticity-assisted multiconfiguration” to establish a bridge between aromaticity and multiconfiguration. Its unique feature lies in that the multiconfiguration characteristics of the unpaired electrons on actinides are mainly assisted by the aromatic skeleton, and the unpaired electrons have no significant effect on the aromaticity.

### Computational details

Geometry optimizations were performed with the PBE0 ^51^ functional augmented with Grimme's D3 dispersion corrections^52^ in Gaussian 16.^53^ In terms of basis sets, a small-core fully relativistic effective core potential (ECP60MDF)^54^ and associated segmented valence basis sets were adopted for actinides and the def2-TZVP basis set was used for main group elements. In the following discussion, the combination of these basis sets is simply labelled as ECP60MDF.

Molecular orbital (MO) based multi-reference methods, including complete active space self-consistent field (CASSCF) and corrections from second-order perturbation theory (CASPT2) along with multi-state CASPT2 (MS-CASPT2),^55–57^ were performed with the all-electron basis set ANO-RCC^58,59^ and the OpenMolcas software.^60^ The scalar relativistic effect was considered through the Douglas–Kroll–Hess (DKH) Hamiltonian,^61,62^ while the spin–orbit (SO) coupling was added as *a posteriori* correction to the MS-CASPT2 energy. Since the scalar relativistic effect can be introduced by relativistic effective core potentials, we also performed CASSCF/CASPT2/MS-CASPT2 calculations with the ECP60MDF basis set. The energy trends obtained with the ANO-RCC basis set together with the DKH correction are completely identical to those with the ECP60MDF basis set (see details in Table S1†). Consequently, the VB self-consistent field (VBSCF)^63^ calculations and corrections from the second-order perturbation theory (VBPT2)^64^ were performed with the ECP60MDF basis set by using the XMVB software.^65,66^ The data listed in the following section are taken from the calculations at the MS-CASPT2/ANO-RCC level unless specified.

It should be noted that the AdNDP, QTAIM and MCI analyses in this work were performed at the PBE0/ECP60MDF theoretical level for the highest spin states, as the DFT analyses yielded identical results with the multi-reference CASSCF method. As for the NICS and current density analyses, we can only perform PBE0/ECP60MDF calculations at this stage. Since the results for the singlet states are comparable to those for the high-spin state in U_2_N_2_, Pa_2_N_2_ and Th_2_N_2_, it is reliable to assess the aromaticity for An_2_N_2_ systems at the DFT level. In all DFT calculations, a tight SCF convergence criterion was imposed.

## Results and discussion

### Multiconfigurational nature of actinide nitrides

**Fig. 2:**
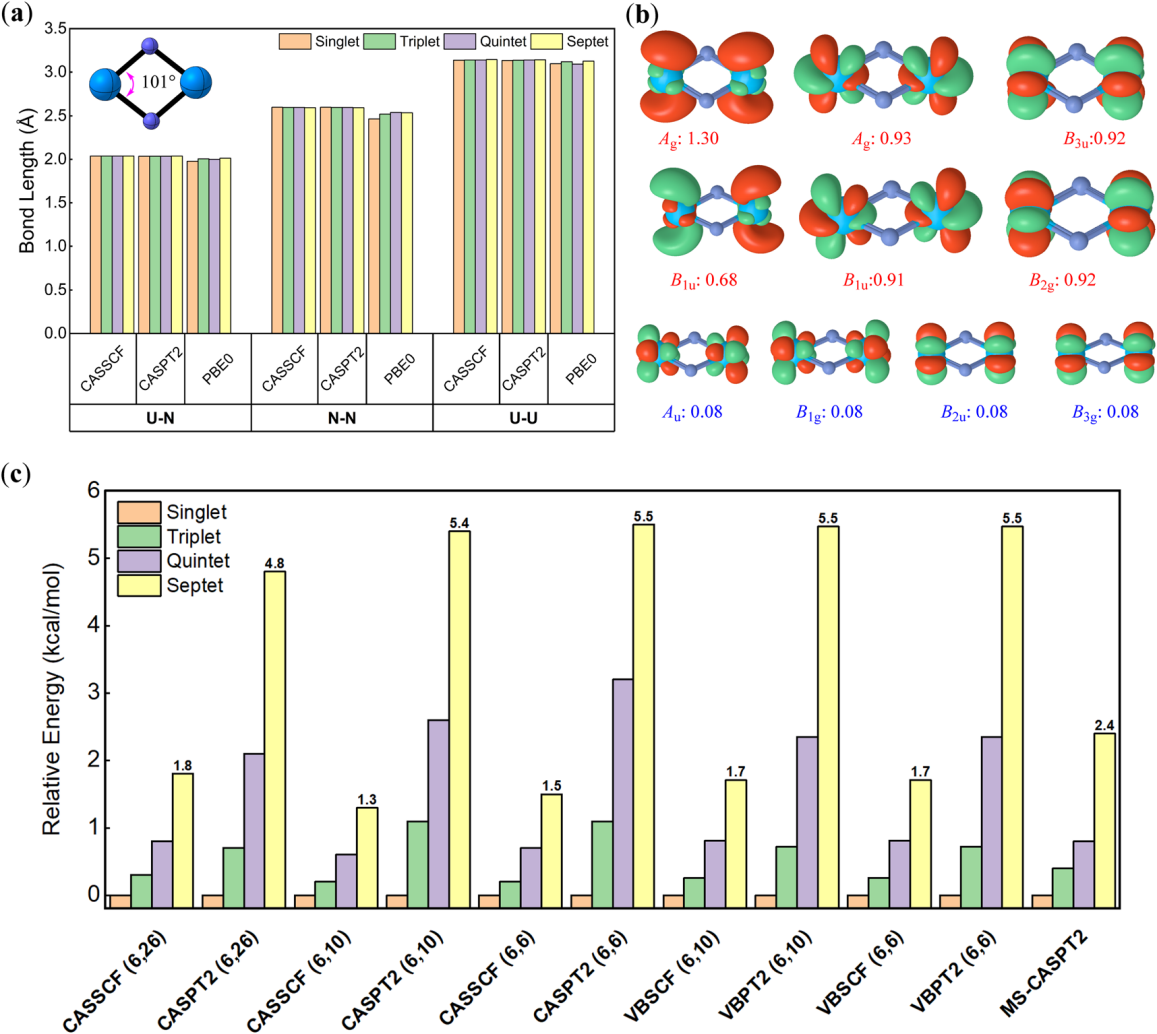
(a) Key bond lengths for optimal U_2_N_2_ with *D*_2h_ symmetry; (b) ten major active orbitals used in multi-reference calculations and their occupation numbers for the state ^1^*A*_g_; (c) relative energies for each spin multiplicity.

The optimal naked U_2_N_2_ (structural data in Table 1) exhibits a rhombic structure with *D*_2h_ symmetry as illustrated in Fig. 2a, in accordance with previous experimental and computational findings.^30,67^ Furthermore, we observed a noteworthy resemblance between the geometry of U_2_N_2_ and the double Möbius aromatic species Pa_2_B_2_, where the total 16 valence electrons form four identical localized Pa–B covalent bonds, two delocalized σ bonds and two delocalized π bonds. For the neutral U_2_N_2_ with 22 valence electrons, we anticipate that 16 valence electrons engage in the same bonding pattern as Pa_2_B_2_, while the remaining 6 electrons are localized on two uranium atoms. The ground state of U_2_N_2_ is predicted to be a septet^30^ or quintet^67^ at the DFT level, but experimental observations indicate that antiferromagnetic U_2_N_2_ nitrides should have a singlet ground state.^29,68^ This discrepancy may arise from the multiconfigurational nature of U_2_N_2_.

**Table 1:** Optimal bond lengths (in Å) for An_2_N_2_ at the PBE0/ECP60MDF level and their Wiberg bond order. The italic data are obtained from the XRD experiments

Species	Bond lengths				Wiberg bond order			
	An–N	An–N	An–An	N–N	An–N	An–N	An–An	N–N
U_2_N_2_	2.013	2.013	3.126	2.536	2.206	2.206	1.391	0.138
Pa_2_N_2_	2.029	2.029	3.145	2.563	2.164	2.164	1.606	0.130
Th_2_N_2_	2.069	2.069	3.179	2.648	2.064	2.064	1.424	0.113
Ac_2_N_2_	2.177	2.177	3.325	2.811	2.025	2.025	1.574	0.078
[U_2_N_2_]^+^	1.996	1.996	3.087	2.531	1.921	1.906	1.210	0.073
Mol I	2.051	2.058	3.288	2.466	2.032	2.001	1.009	0.108
	*2.077*	*2.098*	*3.355*	*2.485*				
Mol II	2.006	2.080	3.252	2.476	2.249	1.904	1.053	0.108
	*2.023*	*2.101*	*3.296*	*2.479*				
Mol III	2.140	2.179	3.381	2.688	1.763	1.617	0.683	0.069
	*2.152*	*2.208*	*3.404*	*2.725*				

To identify the multiconfigurational nature, we resorted to high-level multi-reference methods, in which the 6 valence electrons on metal centres were chosen as the active electrons according to previous theoretical studies.^30^ Standard CASSCF and CASPT2 calculations with 26 active orbitals were first performed for each spin multiplicity and space symmetry. As shown in Fig. 2b, only ten orbitals have occupation numbers higher than 0.01, in which six of them are prominent for all cases. Subsequently, we continued to run the CASSCF and CASPT2 calculations with (6, 10) and (6, 6) active spaces. The computed absolute and relative energies from these two small active spaces are nearly identical and comparable to those from the large (6, 26) active space. Furthermore, multi-state CASPT2 (MS-CASPT2) calculations are conducted based on CASSCF(6, 10) wavefunctions averaged over the first ten states. Remarkably, all multi-reference methods consistently suggest that the ground state of U_2_N_2_ is a singlet, though the energy gaps to the triplet (0.4 kcal mol^−1^), quintet (0.8 kcal mol^−1^) and septet (2.4 kcal mol^−1^) states are trivial, and excitations can easily occur under ambient conditions. For each spin multiplicity, the lowest-lying electronic states are ^1^*A*_g_, ^3^*B*_1u_, ^5^*A*_g_ and ^7^*B*_1u_, respectively, with similar electronic configurations for active space. In particular, the six major orbitals have occupation numbers close to unity, primarily originating from 7s and 5f atomic orbitals of uranium. Moreover, the spin–orbital coupling induced by the six active electrons was computed as negligible. Thus, it can be concluded that the six active electrons are spin-free and strictly localized on metal centres, resulting in the multiconfigurational nature of U_2_N_2_.

**Fig. 3:**
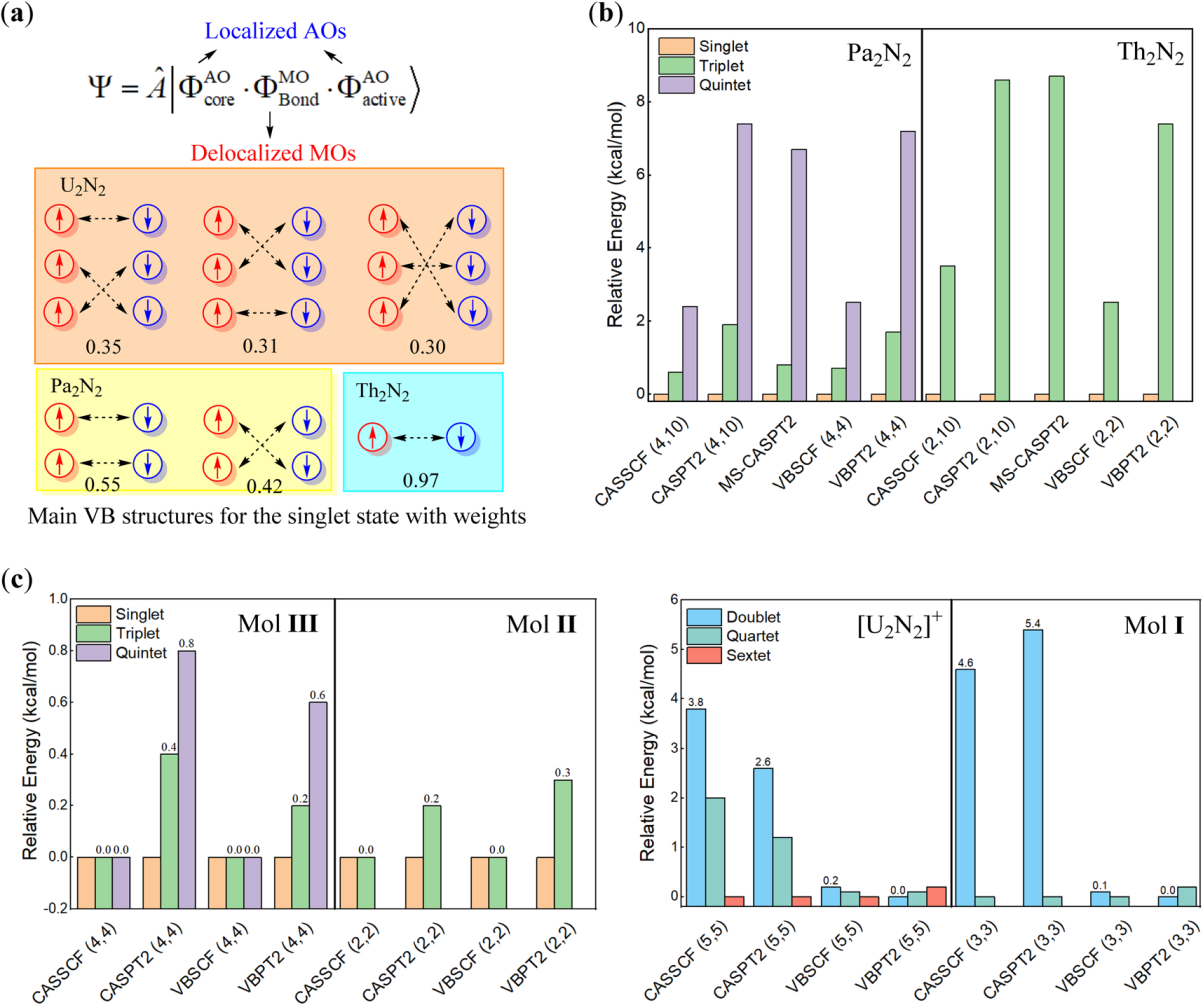
(a) The definition of the VBSCF wavefunction and computed structural weights for major VB structures, and relative energies for (b) theoretically modelled An_2_N_2_ (An = Pa, Th) and (c) [U_2_N_2_]^+^ and experimentally identified compounds I–III with reduced numbers of active electrons and orbitals.

We further conducted VBSCF and VBPT2 calculations within the (6, 10) and (6, 6) active spaces with the ECP60MDF basis set as a supplement. As depicted in Fig. 3a, the VB wavefunction comprises three components, including the core and active orbitals which are expressed with pure atomic orbitals (AOs), and the bonding orbitals which are molecular orbitals (MOs) delocalizing over the whole system. Thus, the above MO- and present VB-based calculations differ in active orbitals. However, the energy gaps from the VBSCF (6, 6)/(6, 10) computations are even closer to those from CASSCF(6, 26) than those from CASSCF(6, 6)/(6, 10), further confirming that the active electrons prefer to localize on metal centres. In VBSCF calculations, the most important VB structure typically involves electron pairings between compatible atomic orbitals, when any chemical bonding is forged between metal centres in U_2_N_2_. However, the major VB structures in the singlet ground state reveal that the active electrons even pair crossing d or f atomic orbitals with different symmetries (as seen in Fig. 3a), indicating that the active electrons are essentially independent of any meaningful bonding between metal centres. Consequently, the active electrons are oriented in opposite directions on each metal centre, giving rise to open-shell singlet ground states. This obviously explains the insignificant energy gaps among different spin states.

As a single-reference method, conventional DFT obviously predicts a septet ground state for U_2_N_2_ with significantly exaggerated energy gaps to the singlet (65.1 kcal mol^−1^), triplet (35.0 kcal mol^−1^) and quintet (25.7 kcal mol^−1^) states at the PBE0/ANO-RCC-DKH level, respectively. Despite the much large energy gaps compared to multi-reference methods, DFT produces nearly identical optimal geometries for each spin multiplicity (see Fig. 2a), suggesting that the arrangement of the spin-free electrons has little impact on the framework of U_2_N_2_. This is further validated by the consistent optimal geometries for each spin multiplicity using either CASSCF/CASPT2(6, 10) or (6, 6) methods. Given that DFT methods can provide convincing geometry for the highest spin multiplicity, the PBE0/ECP60MDF level was employed to optimize the geometries for the remaining studied molecules for the sake of efficiency.

The multiconfigurational character of U_2_N_2_ is also observed both in experimentally identified compounds I–III and theoretically modelled [U_2_N_2_]^+^ and An_2_N_2_ (An = Pa, Th and Ac) clusters. There is no doubt that the optimal geometries for I–III are well consistent with their solid-state molecular structures (*C*_i_ symmetry) determined by X-ray diffraction. Therefore, multi-reference calculations were performed for An_2_N_2_ (An = Pa, Th) with *D*_2h_ symmetry, while [U_2_N_2_]^+^ and compounds I–III adopted *C*_i_ symmetry in calculations. Due to the high computational costs associated with compounds II and III, multi-reference calculations were performed on model systems, where *t*-butyl groups and Pr_i_^3^ groups were replaced with hydrogens. It should be noted that the model systems were also optimized at the PBE0/ECP60MDF level with the U_2_N_2_ core fixed at their crystal structures.

As depicted in Fig. 3c, computational results reveal an open-shell singlet ground state for Pa_2_N_2_, Th_2_N_2_, and compounds II and III, confirming the antiferromagnetic nature of II and III.^29,68^ Even closed-shell singlet Ac_2_N_2_ also exhibits the rhombic structural geometry albeit without any spin-free electrons. In sharp contrast, the ground state of [U_2_N_2_]^+^ and I are found to be sextet and quartet states by CASSCF, VBSCF and CASPT2 methods, while the VBPT2 computations showed that the low spin states are more stable. It is interesting that the energy gaps among different states are less than 1 kcal mol^−1^ for [U_2_N_2_]^+^ and compounds I–III, even lower than the respective gaps for the neutral An_2_N_2_ cluster. It is also worth noting that the energy gaps for the U_2_N_2_ core of I–III, *i.e.* [U_2_N_2_]^3+^, [U_2_N_2_]^4+^ and [U_2_N_2_]^2+^ are nearly identical to those for their parents.

### Double Möbius aromaticity in actinide nitrides

**Fig. 4:**
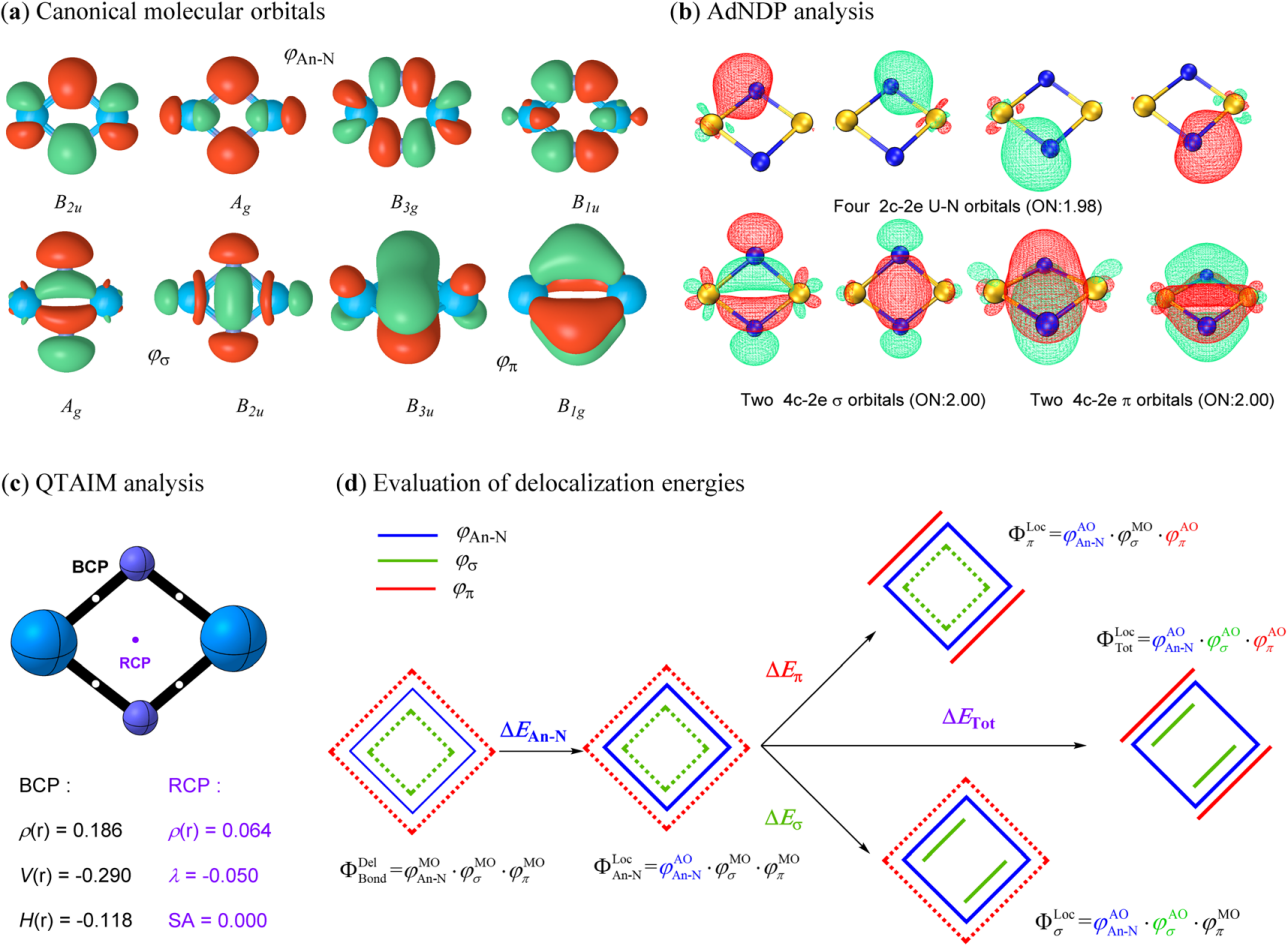
(a) Valence canonical molecular orbitals (CMOs) with an isovalue of 0.05 a.u.; (b) AdNDP analysis showing four localized 2c–2e U–N covalent bonds and four delocalized 4c–2e aromatic bonds along with their occupation numbers (ONs); (c) QTAIM analysis including the electron density *ρ*(r), potential energy density *V*(r), electron energy density *H*(r), electron density curvature (*λ*) and Shannon aromaticity (SA) at BCP (3, −1) or RCP (3, +1) for U_2_N_2_; (d) evaluation of delocalization energies for the chemical bonding of interest.

The above analyses demonstrate that all studied actinide nitrides share a similar planar geometry with equivalent An–N bonds, implying an intrinsic bonding pattern for the An_2_N_2_ rings. In addition to the 6, 4, 2 and 0 active electrons on the metal centers of U, Pa, Th and Ac, respectively, the other 16 valence electrons collectively form eight identical canonical molecular orbitals (CMOs) for each spin multiplicity of all studied An_2_N_2_ clusters (Fig. 4a for U_2_N_2_ and Fig. S1–S7† for the remaining systems). In other words, the bonding orbitals essentially remain invariant despite variations in the number and orientation of the spin-free electrons. The adaptive natural density partitioning (AdNDP)^69^ method provides further insights. Four of the CMOs are found to be a linear combination of four identical 2c–2e An–N covalent bonds, while the remaining CMOs represent two delocalized 4c–2e σ bonds and two delocalized π bonds. It should be noted that there is one delocalized MO with Hückel topology (*B*_2u_ and *B*_3u_) and one with Möbius topology (*A*_g_ and *B*_1g_) for both σ and π systems, indicating a hybrid Hückel–Möbius character.^70–72^ However, the four-membered 4σ/4π An_2_N_2_ with equivalent An–N bonds obviously satisfy the 4n Möbius rule, predominantly demonstrating the double Möbius aromaticity. This is also consistent with the well-identified 4π Möbius aromatic ReB_4_.^73^ Furthermore, the various aromaticity criteria investigated in the following section further endorsed the double Möbius aromaticity in An_2_N_2_.

It becomes clear that the unprecedent double Möbius aromaticity strongly stabilizes the framework of actinide nitrides, allowing the unpaired electrons to be spin-free and either localize on metal centres or bond with ligands. Since the unpaired electrons do not form any bonding interactions among themselves, they do not contribute to the bond orders. Therefore, the Wiberg bond orders (see Table 1) for An–N (*ca.* 2), An–An (*ca.* 1–1.5) and N–N (*ca.* 0.1) primarily come from the bonding orbitals. According to the AdNDP analysis, however, the bond order for An–N should be around 3, while chemical bonding is absent between two actinide atoms. This disagreement may result from the cyclic delocalization in actinide nitrides. To further validate the cyclic delocalization, we employed the quantum theory of atoms in molecules (QTAIM) method^74^ to identify the critical points among bound atoms. As expected, four identical bond critical points (BCPs) are found between adjacent actinide and nitrogen atoms, representing the four localized An–N covalent bonds. In addition, a ring critical point (RCP) precisely at the ring centre suggests that the An–An bonding interaction originates from the cyclic electron delocalization rather than the direct An⋯An communication. Furthermore, the presence of aromaticity in actinide nitrides is supported by the positive electron density, negative electron density curvature and zero Shannon aromaticity value at RCP. We also calculated the multicenter index (MCI)^75^ and found one four-center bond with a MCI (4) value of around 0.5. Besides, there are also four three-center bonds with a MCI (3) value up to 0.7. The MCI results provide support for the presence of the aromaticity in An_2_N_2_.

In pursuit of an improved understanding of the bonding nature, we applied *ab initio* VB methods to quantify the energetics from the electron delocalization effect of the bonding orbitals. In Fig. 4d and the following definition, only the *Φ*_Bond_ components are depicted, while the core and active orbitals remain the same as in the previous definition. We first constructed a localized state (*Φ*^Loc^_An–N_) with the four An–N covalent bonds strictly localized on the corresponding atoms. The energy change (Δ*E*_An–N_) from this localized state to the delocalized *ψ*^Bond^_Del_ state measures the degree of electron delocalization among the four An–N covalent bonds. Subsequently, strictly σ- and π-localized states (*ψ*^Loc^_σ_ and *ψ*^loc^_π_) were established by further localizing two σ and two π orbitals on two adjacent actinide and nitrogen atoms separately. Finally, all eight chemical bonds including four An–N covalent bonds, two delocalized σ bonds and two delocalized π bonds, were localized as in the previous definition to construct a completely localized state (*ψ*^Tot^_Loc_). As a result, the electron delocalization induced by σ and π orbitals, and their combined effect was evaluated based on the energy differences of the σ-, π-, or totally localized states with reference to the *Φ*^Loc^_An–N_ state, respectively. This comprehensive energy analysis offers a thorough examination of the electron delocalization effects in the bonding interactions of interest.

The computational delocalization energies with the VBSCF and VBPT2 methods are identical for each spin multiplicity of actinide nitrides (see Table S2†), again indicating the insignificant effect of active electrons on the planar framework. Therefore, we only collect the data for their ground states at the VBSCF level in Table 2 and VBPT2 level in Table S2.† The minor values of Δ*E*_An–N_ confirm that the four An–N covalent bonds are inherently localized. Remarkably, the energetic gains for the delocalized σ and π components reaches up to around 40 and 30 kcal mol^−1^, respectively, while the total gains amount to 70 kcal mol^−1^. For Ac_2_N_2_, we can also conduct block-localized wavefunction (BLW) computations at the PBE0 level, which is the simplest variant of VB theory and incorporates the efficiency of MO theory.^76–79^ The computed delocalized energies at the PBE0 level are very close to those with the VBSCF method. The high delocalization energies for bonding orbitals are fully consistent with the AdNDP and QTAIM analysis, confirming the delocalization nature of two σ and two π orbitals.

**Table 2:** Computed delocalization energies and extra cyclic resonance energies (ECRE) at the VBSCF level (kcal mol^−1^), the NICS(1)_*zz*_ values (in ppm), the multicenter index (MCI) and integrated induced ring-current (*J*^int^, in *n*A/T) passing the An–N bond by PBE0/ECP60MDF for the highest spin state

Species	Δ*E*_An–N_	Δ*E*_π_	Δ*E*_σ_	Δ*E*_Tot_	ECRE_π_	ECRE_σ_	ECRE_Tot_	NICS(1)_*zz*_	MCI (4)	MCI (3)	*J* ^int,^ b
U_2_N_2_	2.0	41.9	32.9	77.0	41.5	32.5	76.3	−84.7	0.485	0.687	26.4 (23.7)
Pa_2_N_2_	1.9	42.6	33.3	77.9	42.3	33.0	77.3	−46.0	0.478	0.693	23.8 (30.4)
Th_2_N_2_	1.9	40.5	30.2	72.7	40.2	29.9	72.1	−8.9	0.445	0.697	12.6 (13.6)
Ac_2_N_2_^a^	2.0 (2.5)	30.2 (35.0)	21.6 (25.7)	53.1 (61.5)	29.9 (34.5)	21.3 (25.2)	52.6 (60.4)	−9.9	0.382	0.684	10.0
[U_2_N_2_]^+^	2.2	45.8	35.4	83.7	45.4	35.1	82.9	−97.6	0.478	0.680	36.9
Mol I	3.0	49.8	38.4	90.9	49.6	38.2	90.4	−35.7	0.491	0.653	—
Mol II	3.7	45.7	34.4	81.8	45.4	34.1	81.0	−53.7	0.507	0.726	—
Mol III	1.8	45.2	32.4	80.1	45.0	32.2	79.7	−20.1	0.439	0.701	—

a The data in parenthesis are obtained with the BLW method at the PBE0 level.

b The data in parenthesis refer to the singlet state.

However, we understood that aromaticity refers to the “extra” stability in a cyclic system relative to non-cyclic systems. In this regard, extra cyclic resonance energy (ECRE), defined as the delocalization energy difference between a cyclic compound and its appropriate acyclic reference, serves as a convincing criterion for assessing the aromaticity.^80,81^ Specifically, the value of positive ECRE indicates the magnitude of aromaticity. Herein, linear [An–N–An–N] and [H_2_N–An–N–An–NH] were considered as the acyclic references to evaluate ECRE values, as they possess the same number of delocalized electrons compared to cyclic An_2_N_2_. Besides, linear An_2_N_2_ has the same number of atoms as cyclic An_2_N_2_, while An_2_N_3_H_3_ has the same number of An–N bonds as cyclic An_2_N_2_. Linear An_2_N_2_ is obviously less stable than cyclic An_2_N_2_ due to the loss of one An–N bond and the linear constraint, but this is not an issue here. Notably, the delocalization energies for both acyclic references are negligible, *e.g.* 0.4 kcal mol^−1^ for each component and 0.7 kcal mol^−1^ for the total contribution in U_2_N_3_H_3_. This is also reflected by the CMOs in linear systems (see Fig. S8†), where the canonical σ and π orbitals in the cyclic system become relatively localized on the adjacent actinide and nitrogen atoms. In other words, the concerned An–N bonds in linear systems can be viewed as triple bonds like in acetylene. Accordingly, the ECREs relative to An_2_N_3_H_3_ are listed in Table 2. The significant positive ECREs strongly authenticate that the actinide nitrides represent a new family of double Möbius aromatic compounds.

We further calculated the nuclear-independent chemical shift (NICS)^82^ values to examine the magnetic shielding effects of the delocalized electrons, and the *zz* component of NICS above the ring center is considered in this work because it was believed to be a more suitable choice for assessing aromaticity for planar rings.^83^ The negative NICS(1)_*zz*_ values are consistent with the positive ECREs, confirming the aromaticity in An_2_N_2_.

**Fig. 5:**
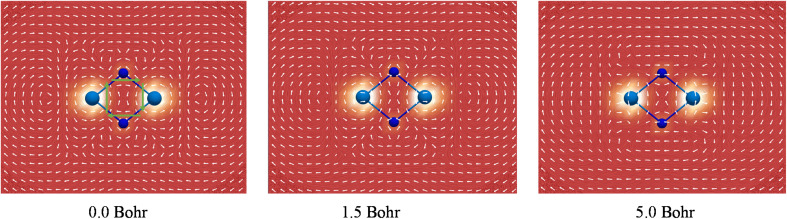
The strength and direction of the induced current of U_2_N_2_ at 0.0, 1.5 and 5.0 Bohr above the molecular plane calculated by the GIMIC program. Clockwise currents represented by green arrows in the inner of U_2_N_2_ ring are diatropic and indicate aromaticity.

It has been well recognized that the NICS has limitations for assessing aromaticity in heavy metal clusters^84–88^ and even in organic molecules.^89^ In this regard, the negative NICS values for Ac_2_N_2_ may just be a coincidence. In contrast, the global diamagnetic induced ring-current is believed to be a more reliable criterion for aromaticity in heavy metal clusters,^90–93^ and has been well-validated in a comprehensive theoretical work by Orozco-Ic *et al.* very recently.^94^ Therefore, we performed rigorous ring-current analyses. Fig. 5 depicts the line integral convolution visualization of the induced currents by the gauge including magnetically induced current (GIMIC) method^95–97^ for U_2_N_2_ (see Fig. S9† for other systems). There are diatropic ring-currents inside and outside of the ring, which become much clearer at 5.0 Bohr above the molecular plane. It is also obvious that the paramagnetic currents induced by core electrons on metal centers have a significant effect on the inner and outer ring-currents in U_2_N_2_, in agreement with the findings by Orozco-Ic *et al.*^94^ In order to eliminate the effect of core electrons, we calculated the induced current by setting the end point of the crossing plane at the midpoint of the An–N bond,^98^ which can better reflect the strength of the inner ring-current. As listed in Table 2, the results indicate that An_2_N_2_ is magnetically aromatic, which is comparable to and even stronger than the aromaticity in benzene with a *J*^int^ value of around 12 *n*A/T. Besides, the isosurfaces of anisotropy of the induced current density (ACID)^99,100^ generated by all CMOs, two delocalized π and two delocalized σ, provide further support for the presence of diatropic inner ring-currents in An_2_N_2_ (see Fig. S10†).

## Conclusions

In conclusion, comprehensive chemical bonding analyses show that the framework of actinide nitrides (An_2_N_2_) adopts a planar geometry with four delocalized σ and four delocalized π electrons, satisfying the 4*n* Möbius aromaticity rule. Furthermore, the presence of double Möbius aromaticity supports actinide nitrides to exhibit multiconfigurational character due to the unpaired electrons on metal centers, namely the “aromaticity-assisted multiconfiguration” (AAM) bonding model. Since the unpaired electrons are spin-free to strictly localize on the metal centers or form chemical bonding with ligands, the energy gaps between different spin states are remarkably minor. Nevertheless, the actinide nitrides always have an open-shell singlet ground state, except when the number of unpaired electrons is odd.

## Author contributions

X. H. L., Y. R. M. and W. W. directed the project. X. H. L. conceived the idea, and designed, and performed the calculations. X. H. L. and Y. R. M. wrote the manuscript. X. X. L. conducted the ring current analysis. All authors discussed the results and commented on the manuscript.

## Conflicts of interest

There are no conflicts to declare.

## Supplementary Material

SC-015-D4SC01549E-s001
