# Supplementary material for: Multiconfigurational actinide nitrides assisted by double Möbius aromaticity
Source: Chem Sci. 2024 Apr 26;15(21):8216–26. doi: 10.1039/d4sc01549e (PMC11134321; doi:10.1039/d4sc01549e)
Supplement: SC-015-D4SC01549E-s001 [file SC-015-D4SC01549E-s001.pdf]

Electronic Supplementary Material (ESI) for Chemical Science.
This journal is © The Royal Society of Chemistry 2024

Supplementary Information for

Multiconfigurational Actinide Nitrides Assisted by Double Möbius Aromaticity

Xuhui Lin*[1], Xiaoli Lu[2], Shenghui Tang[2], Wei Wu[3*], Yirong Mo*[4]

[1] School of Physics, Central South University, Changsha, Hunan 410083, China;

[2] School of Chemistry, Southwest Jiaotong University, Chengdu, Sichuan 610031, China;

[3] The State Key Laboratory of Physical Chemistry of Solid Surfaces, iChEM, Fujian Provincial Key Laboratory of Theoretical and Computational Chemistry and College of Chemistry and Chemical Engineering, Xiamen University, Xiamen, Fujian 361005, China;

[4] Department of Nanoscience, Joint School of Nanoscience and Nanoengineering, University of North Carolina at Greensboro, Greensboro, NC 27401, United States.

E-mails:

xuhui.lin@csu.edu.cn (XL); weiwu@xmu.edu.cn (WW); y_mo3@uncg.edu (YM)

Table of Contents

Table S1. Energy gaps between different spin states with ANO-RCC basis set with DKH correction and ECP60MDF basis set for U_2N_2 3

Table S2. Computed delocalization energies for each spin multiplicity at VBSCF and VBPT2 level 4

Table S3. Major optimal parameters for U_2N_2 by various methods......................5

Table S4-S7. Computed absolute energies and relative energies of all electronic states for singlet, triplet, quintet and septet U_2N_2 using a (6,10) active space with ANO-RCC-DKH basis set...6

Figure S1-S7. Eight Valence canonical molecular orbitals (CMOs) with the isovalue of 0.045 a.u. for Pa_2N_2, Th_2N_2, Ac_2N_2, $[U_2N_2]^+$ and compounds **I-III**.....................10

Figure S8. The canonical molecular orbitals (CMOs) with the isovalue of 0.05 a.u. for linear U_2N_2 and [H_2N–An–N–An–NH] relative to the delocalized σ and πorbitals in cyclic U_2N_2 .... .13

Figure S9. The strength and direction of the induced current for Pa_2N_2, Th_2N_2 and Ac_2N_2 by GIMIC program........... 14

Figure S10. Isosurfaces of ACID (isovalue = 0.03) for U2N2, Pa_2N_2, Th_2N_2 and Ac_2N_2..15

Optimal Coordinates xyz for all studied systems...16

Table S1. Energy gaps between different spin states with ANO-RCC basis set with DKH correction and ECP60MDF basis set for U_2N_2.

Method	Basis set	^1A_g	$^3B_{1u}$	^5A_g	$^7B_{1u}$
PBE0	ECP60MDF	114.7	12.7	23.0	0.0
	ANO-RCC	63.4	25.8	20.7	0.0
CASSCF (6,26)	ECP60MDF	0.0	0.2	0.8	1.7
	ANO-RCC	0.0	0.3	0.8	1.8
CASPT2 (6,26)	ECP60MDF	0.0	0.7	2.0	4.2
	ANO-RCC	0.0	0.7	2.1	4.5
CASSCF (6,10)	ECP60MDF	0.0	0.2	0.6	1.3
	ANO-RCC	0.0	0.2	0.7	1.3
CASPT2(6,10)	ECP60MDF	0.0	1.0	2.8	5.5
	ANO-RCC	0.0	1.0	2.9	6.0
MS-CASPT2(6,10)	ECP60MDF	0.0	0.4	0.7	2.2
	ANO-RCC	0.0	0.4	0.8	2.4
CASSCF (6,6)	ECP60MDF	0.0	0.2	0.7	1.4
	ANO-RCC	0.0	0.2	0.7	1.5
CASPT2 (6,6)	ECP60MDF	0.0	1.2	3.1	6.4
	ANO-RCC	0.0	1.1	3.2	6.5
VBSCF (6,6)	ECP60MDF	0.0	0.3	0.8	1.7
VBPT2 (6,6)	ANO-RCC	0.0	0.7	2.4	5.5

Table S2. Computed delocalization energies for each spin multiplicity at VBSCF and VBPT2 level.

Species	Spin	VBSCF				VBPT2			
		$\Delta E_{U\text{-}N}$	ΔE_{π}	ΔE_{σ}	ΔE_{total}	$\Delta E_{U\text{-}N}$	ΔE_{π}	ΔE_{σ}	ΔE_{total}
U_2N_2	1	2.0	41.9	32.9	77.0	0.8	24.3	20.4	42.4
	3	2.0	42.0	32.9	77.1	0.8	24.4	20.5	42.5
	5	2.0	41.9	33.1	77.1	0.8	24.2	20.8	42.5
	7	2.0	41.9	33.2	77.1	0.8	23.9	21.1	42.3
Pa_2N_2	1	1.9	42.6	33.3	77.9	0.7	24.3	20.5	42.7
	3	1.9	42.6	33.3	77.9	0.7	24.3	20.5	42.7
	5	1.9	42.5	33.3	77.9	0.6	23.9	20.5	42.3
Th_2N_2	1	1.9	40.5	30.2	72.7	0.9	21.6	16.8	36.2
	3	1.9	40.4	30.2	72.7	0.8	21.5	16.8	36.1
Ac_2N_2	1	2.0	30.2	21.6	53.1	1.5	11.5	15.2	25.6
$[U_2N_2]^+$	2	2.2	45.6	35.2	83.6	0.8	26.5	23.7	47.9
	4	2.2	45.7	35.3	83.6	0.8	26.5	24.2	47.8
	6	2.2	45.8	35.4	83.7	0.8	26.7	24.7	48.1
Mol I	2	2.9	49.8	38.3	90.9	1.9	32.5	32.8	60.1
	4	3.0	49.8	38.4	90.9	1.9	32.4	32.8	60.0
Mol II	1	3.7	45.7	34.4	82.7	3.0	28.3	28.2	50.7
	3	3.7	45.7	34.4	82.7	3.0	28.1	28.0	50.4
Mol III	1	1.8	32.4	45.2	80.1	0.8	23.8	28.1	47.4
	3	1.8	32.4	45.2	80.1	0.9	23.8	28.1	47.2
	5	1.8	32.4	45.2	80.1	0.8	23.8	27.8	47.0

Table S3. Major optimal parameter for U2N2 by PBE0/ECP60MDF and CASSCF(CASPT2)/ANO-RCC-DKH.

Method	Spin	U-U	U-N	N-N	$\theta_{N\text{-}U\text{-}N}$
PBE0	1	3.097	1.979	2.466	77.1
	3	3.120	2.006	2.521	77.9
	5	3.091	2.000	2.540	78.8
	7	3.126	2.013	2.536	78.1
CASSCF(6,10)	1	3.137	2.037	2.599	79.3
	3	3.138	2.037	2.598	79.2
	5	3.140	2.037	2.597	79.2
	7	3.144	2.038	2.595	79.1
CASSCF(6.6)	1	3.131	2.035	2.601	79.4
	3	3.133	2.036	2.600	79.4
	5	3.135	2.036	2.599	79.3
	7	3.139	2.037	2.597	79.2
CASPT2(6,10)	1	3.133	2.035	2.599	79.4
	3	3.136	2.036	2.597	79.3
	5	3.139	2.036	2.596	79.2
	7	3.142	2.037	2.594	79.1
CASPT2(6.6)	1	3.128	2.034	2.601	79.5
	3	3.129	2.034	2.601	79.5
	5	3.134	2.035	2.598	79.3
	7	3.138	2.036	2.596	79.2

Table S4. Computed absolute energies (AE, a.u.) and relative energies (RE, kcal/mol) of all electronic states for singlet U_2N_2 using a (6,10) active space with ANO-RCC-DKH basis set.

State	CASSCF		CASPT2	
	AE	RE	AE	RE
^1A_g	-56008.6780	0.0	-56010.1106	0.0
$^1B_{3u}$	-56008.6611	10.6	-56010.1031	4.7
$^1B_{2u}$	-56008.6629	9.5	-56010.1051	3.5
$^1B_{1g}$	-56008.6758	1.4	-56010.1116	-0.6
$^1B_{1u}$	-56008.6533	15.5	-56010.0986	7.6
$^1B_{2g}$	-56008.6612	10.6	-56010.1032	4.6
$^1B_{3g}$	-56008.6629	9.5	-56010.1049	3.6
^1A_u	-56008.6758	1.4	-56010.1117	-0.7

Table S5. Computed absolute energies (AE, a.u.) and relative energies (RE, kcal/mol) of all electronic states for triplet U_2N_2 using a (6,10) active space with ANO-RCC-DKH basis set.

State	CASSCF		CASPT2	
	AE	RE	AE	RE
^3A_g	-56008.6628	9.3	-56010.0870	13.8
$^3B_{3u}$	-56008.6608	10.6	-56010.1018	4.5
$^3B_{2u}$	-56008.6625	9.5	-56010.1028	3.9
$^3B_{1g}$	-56008.6754	1.4	-56010.1101	-0.7
$^3B_{1u}$	-56008.6777	0.0	-56010.1090	0.0
$^3B_{2g}$	-56008.6607	10.6	-56010.1017	4.5
$^3B_{3g}$	-56008.6625	9.5	-56010.1029	3.8
^3A_u	-56008.6754	1.4	-56010.1100	-0.7

Table S6. Computed absolute energies (AE, a.u.) and relative energies (RE, kcal/mol) of all electronic states for quintet U_2N_2 using a (6,10) active space with ANO-RCC-DKH basis set.

State	CASSCF		CASPT2	
	AE	RE	AE	RE
^5A_g	-56008.6769	0.0	-56010.1059	0.0
$^5B_{3u}$	-56008.6600	10.6	-56010.0986	4.6
$^5B_{2u}$	-56008.6501	16.9	-56010.0863	12.3
$^5B_{1g}$	-56008.6628	8.8	-56010.0935	7.8
$^5B_{1u}$	-56008.6629	8.8	-56010.0873	11.7
$^5B_{2g}$	-56008.6487	17.7	-56010.0852	12.9
$^5B_{3g}$	-56008.6501	16.8	-56010.0863	12.3
^5A_u	-56008.6747	1.4	-56010.1068	-0.6

Table S7. Computed absolute energies (AE, a.u.) and relative energies (RE, kcal/mol) of all electronic states for septet U_2N_2 using a (6,10) active space with ANO-RCC-DKH basis set.

State	CASSCF		CASPT2	
	AE	RE	AE	RE
$^7\boldsymbol{A_g}$	-56008.6514	15.3	-56010.0900	6.9
$^7\boldsymbol{B_{3u}}$	-56008.6588	10.8	-56010.0944	4.1
$^7\boldsymbol{B_{2u}}$	-56008.6605	9.6	-56010.0950	3.8
$^7\boldsymbol{B_{1g}}$	-56008.6738	1.3	-56010.1025	-1.0
$^7\boldsymbol{B_{1u}}$	-56008.6759	0.0	-56010.1010	0.0
$^7\boldsymbol{B_{2g}}$	-56008.6588	10.7	-56010.0944	4.1
$^7\boldsymbol{B_{3g}}$	-56008.6604	9.7	-56010.0936	4.6
$^7\boldsymbol{A_u}$	-56008.6737	1.4	-56010.1026	-1.0

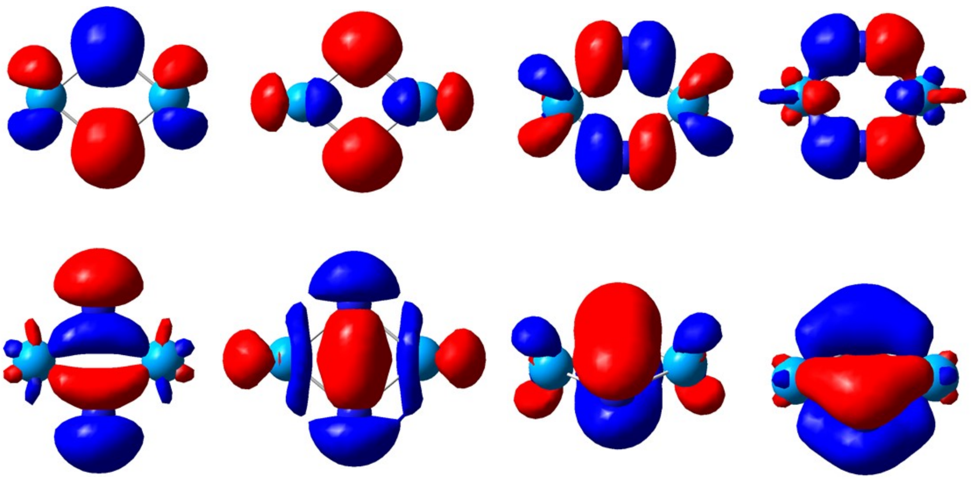

Figure S1. Eight Valence canonical molecular orbitals (CMOs) with the isovalue of 0.05 a.u. for Pa_2N_2.

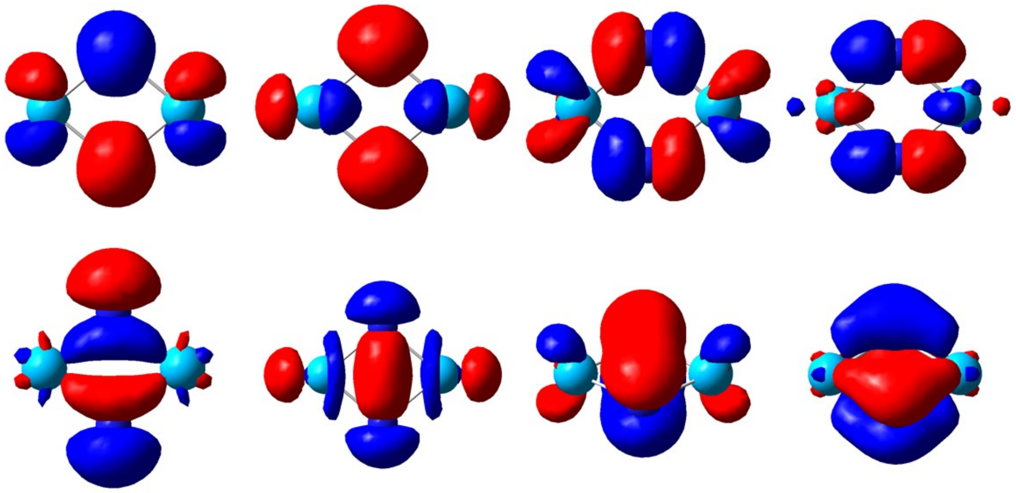

Figure S2. Eight Valence canonical molecular orbitals (CMOs) with the isovalue of 0.05 a.u. for Th_2N_2.

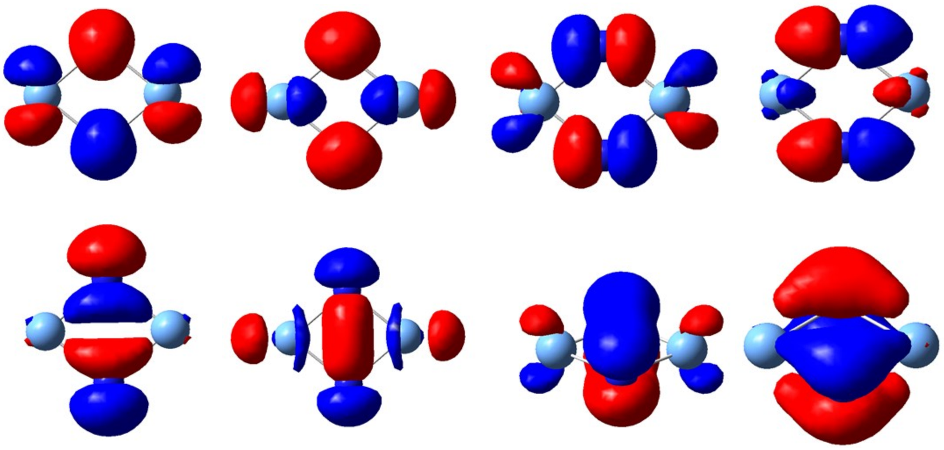

Figure S3. Eight Valence canonical molecular orbitals (CMOs) with the isovalue of 0.05 a.u. for Ac_2N_2.

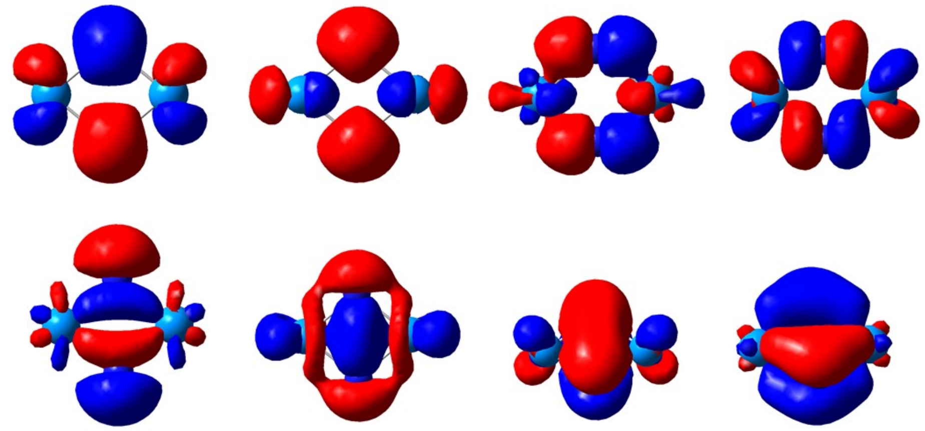

Figure S4. Eight Valence canonical molecular orbitals (CMOs) with the isovalue of 0.05 a.u. for $[U_2N_2]^+$.

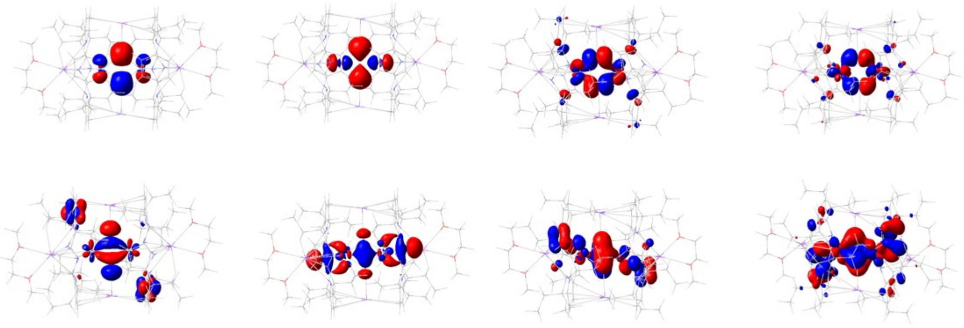

Figure S5. Eight Valence canonical molecular orbitals (CMOs) with the isovalue of 0.04 a.u. for compounds **I**.

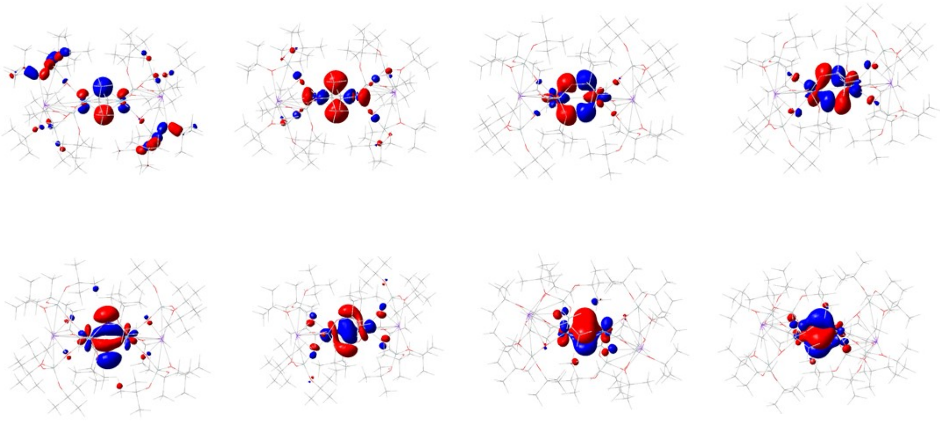

Figure S6. Eight Valence canonical molecular orbitals (CMOs) with the isovalue of 0.04 a.u. for compounds **II**.

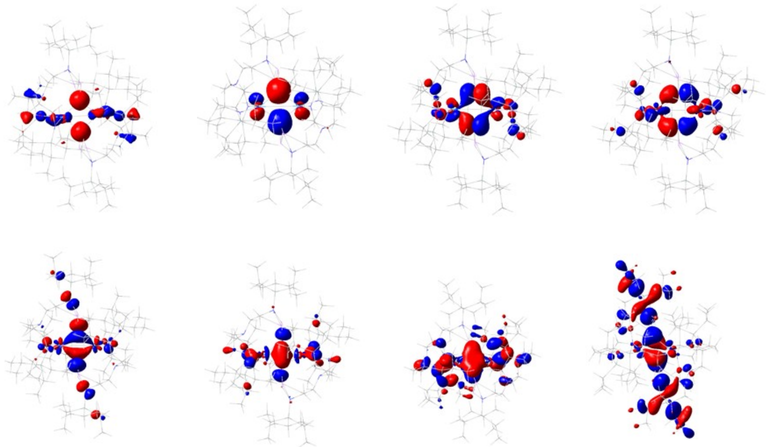

Figure S7. Eight Valence canonical molecular orbitals (CMOs) with the isovalue of 0.04 a.u. for compounds **III**.

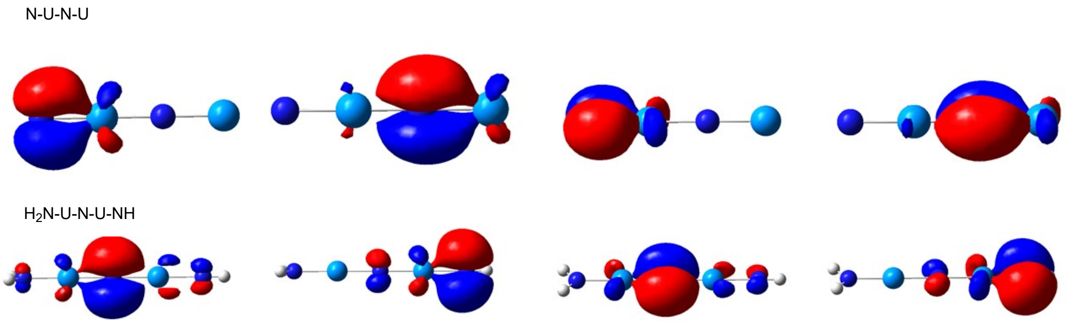

Figure S8. The canonical molecular orbitals (CMOs) with the isovalue of 0.05 a.u. for linear U_2N_2 and [H_2N–An–N–An–NH] relative to the delocalized σ and π orbitals in cyclic U_2N_2.

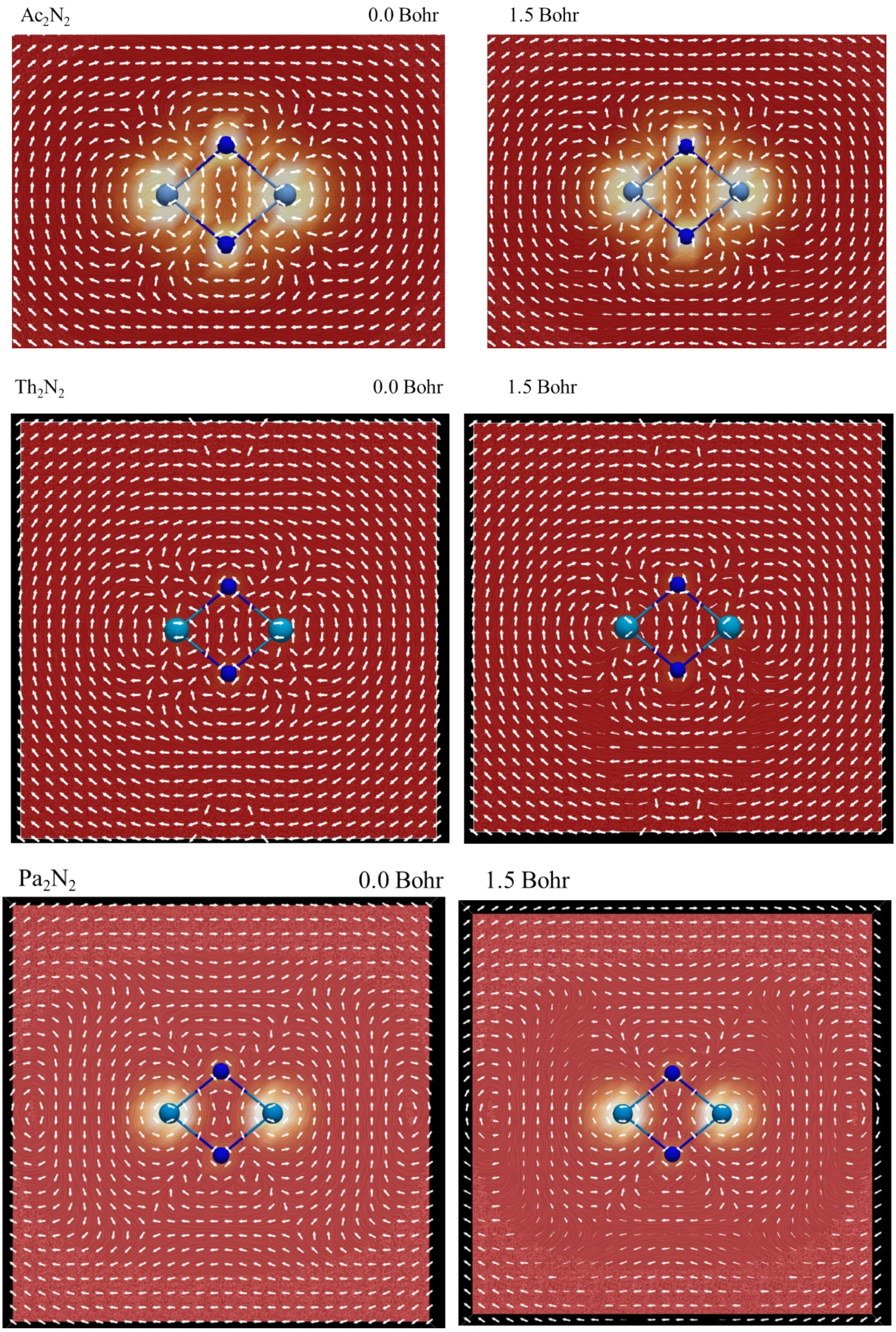

Figure S9. The strength and direction of the induced current for Pa_2N_2, Th_2N_2 and Ac_2N_2 by GIMIC program.

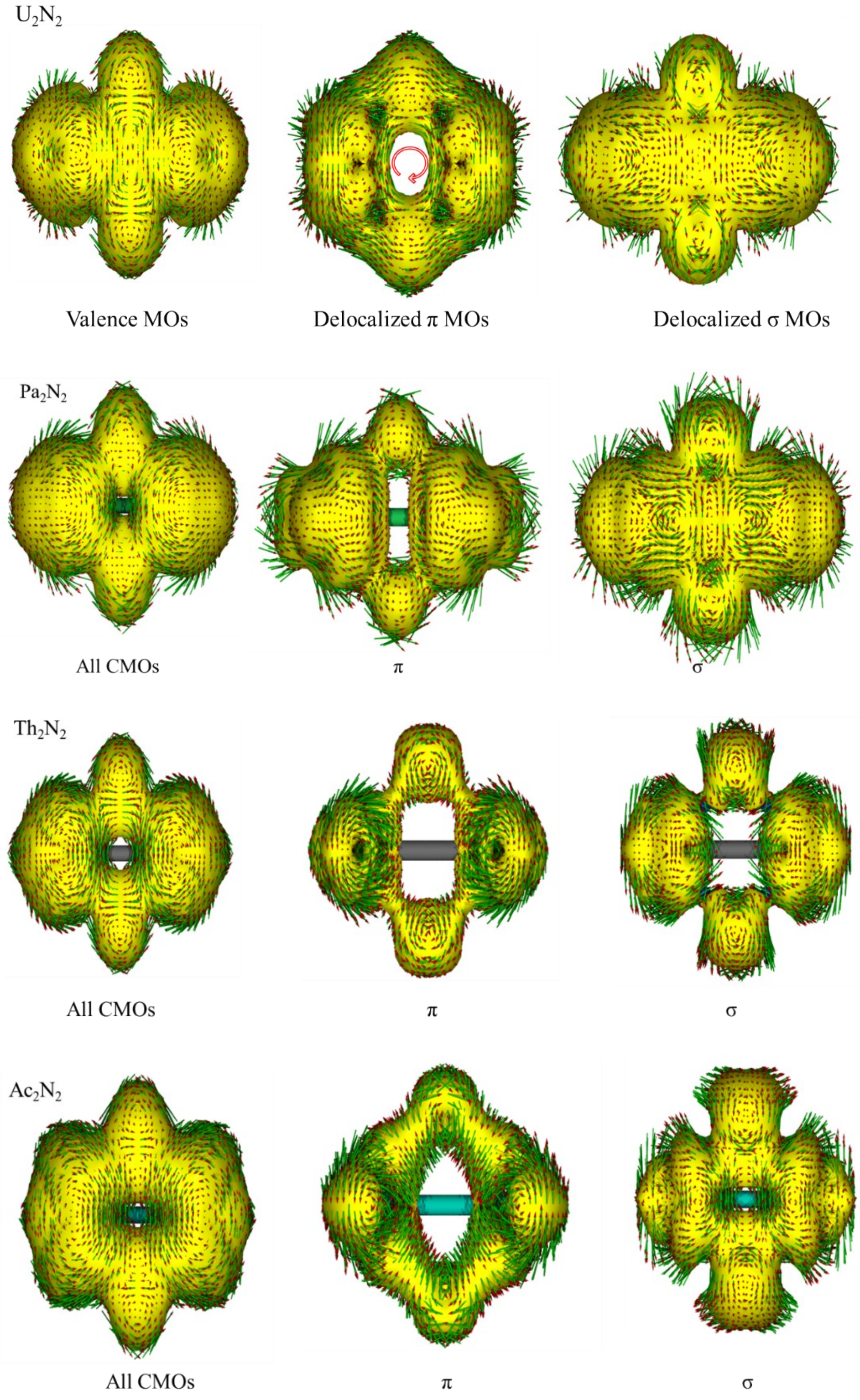

Figure S10. Isosurfaces of AICD (isovalue = 0.03) for Pa_2N_2, Th_2N_2 and Ac_2N_2 generated from all eight CMOs, the two delocalized π MOs, and two delocalized σ MOs.

Optimal geometries for U_2N_2 with CASSCF (6,10)/ANO-RCC

Singlet

U	0.00000000	0.00000000	1.56657001
U	0.00000000	0.00000000	-1.56657001
N	0.00000000	1.29957816	0.00000000
N	0.00000000	-1.29957816	0.00000000

Triplet

U	0.00000000	0.00000000	1.56800149
U	0.00000000	0.00000000	-1.56800149
N	0.00000000	1.29852079	0.00000000
N	0.00000000	-1.29852079	0.00000000

Quintet

U	0.00000000	0.00000000	1.56931707
U	0.00000000	0.00000000	-1.56931707
N	0.00000000	1.29782925	0.00000000
N	0.00000000	-1.29782925	0.00000000

Septet

U	0.00000000	0.00000000	1.57115034
U	0.00000000	0.00000000	-1.57115034
N	0.00000000	1.29722775	0.00000000
N	0.00000000	-1.29722775	0.00000000

Optimal geometries for U_2N_2 with CASSCF (6,6)/ANO-RCC

Singlet

U	0.00000000	0.00000000	1.56409112
U	0.00000000	0.00000000	-1.56409112
N	0.00000000	1.30044046	0.00000000
N	0.00000000	-1.30044046	0.00000000

Triplet

U	0.00000000	0.00000000	1.56441231
U	0.00000000	0.00000000	-1.56441231
N	0.00000000	1.30043270	0.00000000
N	0.00000000	-1.30043270	0.00000000

Quintet

U	0.00000000	0.00000000	1.56681723

U	0.00000000	0.00000000	-1.56681723
N	0.00000000	1.29883034	0.00000000
N	0.00000000	-1.29883034	0.00000000

Septet

U	0.00000000	0.00000000	1.56889426
U	0.00000000	0.00000000	-1.56889426
N	0.00000000	1.29816673	0.00000000
N	0.00000000	-1.29816673	0.00000000

Optimal geometries for U_2N_2 with PBE0/ECP60MDF

Singlet

U	0.00000000	0.00000000	1.54830500
U	0.00000000	0.00000000	-1.54830500
N	0.00000000	1.23281400	0.00000000
N	0.00000000	-1.23281400	0.00000000

Triplet

U	0.00000000	0.00000000	1.55995600
U	0.00000000	0.00000000	-1.55995600
N	0.00000000	1.26057500	0.00000000
N	0.00000000	-1.26057500	0.00000000

Quintet

U	0.00000000	0.00000000	1.54562700
U	0.00000000	0.00000000	-1.54562700
N	0.00000000	1.26980700	0.00000000
N	0.00000000	-1.26980700	0.00000000

Septet

U	0.00000000	0.00000000	1.56313900
U	0.00000000	0.00000000	-1.56313900
N	0.00000000	1.26815300	0.00000000
N	0.00000000	-1.26815300	0.00000000

Optimal geometries for I with PBE0/ECP60MDF

C	5.48850715	3.88554747	2.95706660
C	2.54594820	3.26923591	4.72656221
C	2.49707949	3.53158908	3.22890253
C	3.30331547	4.31682303	0.40483723
C	4.80123965	2.53648247	2.83099772
C	7.04139821	2.93040385	-0.09240642
C	3.32615394	2.56826674	2.35158921
C	3.28659986	4.19194334	-1.00818859
C	3.24669102	3.01685619	0.90867080
C	-3.81354477	3.91644202	3.00244553
C	-1.38929564	2.55601298	4.33626593
C	-5.19072337	4.18949559	2.41415596
C	-2.70583189	4.54109445	0.32192112
C	-1.51547199	2.91594665	2.86545961
C	5.49409399	3.31723739	-3.17681495
C	2.76264408	1.17034672	2.47849804
C	3.22293328	2.82443265	-1.28393603
C	-2.78243313	4.44112816	-1.09265876
C	-2.94780428	2.85564594	2.27590576
C	8.52211622	1.12269509	-0.35180736
C	1.45138486	0.80782667	2.82179191
C	4.06433601	3.03208107	-3.61756343
C	-2.87783084	3.24712298	0.81704299
C	3.17434686	2.19743406	-2.66261643
C	-3.50907477	1.46589569	2.51916851
C	-2.99410200	3.09250871	-1.36876755
C	-4.56448053	1.17496210	3.37858285
C	8.58505074	-0.32302220	-0.77627916
C	1.44896104	-0.59969066	2.91581467
C	1.71785417	2.28571838	-3.19217288
C	2.76077655	-1.00926599	2.63595495
C	-7.93049188	2.48294222	0.12434197
C	-5.50205377	3.73172317	-3.05980674
C	2.81373797	-2.64080002	5.22439545
C	-2.58540596	3.19160651	-3.82599706
C	3.60434059	0.74084530	-2.69681932
C	4.81987179	-2.38095371	2.92788324
C	-3.29057776	2.42451598	-2.68824813
C	-4.62446863	-0.22891972	3.49193338
C	4.62446863	0.22891972	-3.49193338
C	1.49597680	1.74391581	-4.59544105
C	-1.49597680	-1.74391581	4.59544105
C	3.29057776	-2.42451598	2.68824813

C	-4.81987179	2.38095371	-2.92788324
C	7.93049188	-2.48294222	-0.12434197
C	5.50205377	-3.73172317	3.05980674
C	2.58540596	-3.19160651	3.82599706
C	-2.81373797	2.64080002	-5.22439545
C	-3.60434059	-0.74084530	2.69681932
C	-8.58505074	0.32302220	0.77627916
C	-2.76077655	1.00926599	-2.63595495
C	4.56448053	-1.17496210	-3.37858285
C	-1.71785417	-2.28571838	3.19217288
C	2.99410200	-3.09250871	1.36876755
C	-1.44896104	0.59969066	-2.91581467
C	3.50907477	-1.46589569	-2.51916851
C	-3.17434686	-2.19743406	2.66261643
C	-8.52211622	-1.12269509	0.35180736
C	-4.06433601	-3.03208107	3.61756343
C	2.87783084	-3.24712298	-0.81704299
C	-1.45138486	-0.80782667	-2.82179191
C	2.78243313	-4.44112816	1.09265876
C	2.94780428	-2.85564594	-2.27590576
C	-3.22293328	-2.82443265	1.28393603
C	-5.49409399	-3.31723739	3.17681495
C	-2.76264408	-1.17034672	-2.47849804
C	5.19072337	-4.18949559	-2.41415596
C	2.70583189	-4.54109445	-0.32192112
C	1.51547199	-2.91594665	-2.86545961
C	3.81354477	-3.91644202	-3.00244553
C	1.38929564	-2.55601298	-4.33626593
C	-7.04139821	-2.93040385	0.09240642
C	-3.24669102	-3.01685619	-0.90867080
C	-3.28659986	-4.19194334	1.00818859
C	-3.32615394	-2.56826674	-2.35158921
C	-4.80123965	-2.53648247	-2.83099772
C	-3.30331547	-4.31682303	-0.40483723
C	-2.49707949	-3.53158908	-3.22890253
C	-2.54594820	-3.26923591	-4.72656221
C	-5.48850715	-3.88554747	-2.95706660
H	5.03043815	4.51389273	3.73701659
H	1.85305820	3.93733605	5.26187489
H	6.54898238	3.75766745	3.22888041
H	3.55155557	3.43724437	5.14245713
H	2.82758555	4.56306653	3.02883781
H	5.44524908	4.44808626	2.01244766
H	3.39124125	5.24009207	0.97835729

H	2.25972786	2.23253312	4.95839017
H	1.45174548	3.48859565	2.88344366
H	7.40761909	3.12476076	0.93163078
H	4.84035744	2.00417335	3.79538840
H	-3.24101971	4.85969230	3.00562147
H	5.96805503	3.16230459	-0.13902981
H	3.35986887	5.00148491	-1.73568298
H	-1.94511120	3.25280229	4.98210526
H	-2.60971764	5.45706926	0.90757581
H	7.58076615	3.59156389	-0.79602026
H	-5.72534609	4.95065198	3.00618768
H	-1.12434982	3.93649431	2.69536242
H	-0.33781288	2.57918246	4.66036951
H	5.51957917	4.05549238	-2.36463451
H	5.36351236	1.89632089	2.13602838
H	-3.91357747	3.62897081	4.06006428
H	6.08514206	3.71660346	-4.01785865
H	-5.10906455	4.55313115	1.37915813
H	8.90023554	1.22007991	0.68645278
H	-2.75293944	5.26096896	-1.81121808
H	3.55206765	3.99383570	-3.79578314
H	-1.77855565	1.54441031	4.52240051
H	0.61503286	1.46291781	3.02389114
H	5.99636168	2.41123317	-2.80986450
H	-0.89657921	2.23958076	2.25413108
H	-5.80681208	3.27947178	2.40165954
H	9.19367067	1.71254037	-1.00913413
H	-5.18792195	1.88423720	3.91791179
H	1.41182755	3.34859440	-3.15457205
H	-7.65174839	2.76823811	1.15522788
H	4.07846399	2.52478840	-4.59284250
H	-5.28967055	4.36648875	-2.18511944
H	2.54278987	-1.57523564	5.27812173
H	-2.90162267	4.24630435	-3.79065971
H	9.64373243	-0.65186951	-0.75090570
H	5.02280479	-1.75757977	3.81374356
H	-7.25108301	2.99334868	-0.57218598
H	3.86558530	-2.73410584	5.53768936
H	-5.16993148	4.28129895	-3.95507491
H	-1.80098514	-0.68896691	4.65557480
H	-8.96480151	2.82671671	-0.06937051
H	-1.50597103	3.20513090	-3.60576134
H	5.27324033	-1.83122500	2.09257964
H	1.07813696	1.74739476	-2.47393913

H	0.61359156	-1.22403636	3.20037127
H	8.22684728	-0.42246161	-1.82118178
H	5.31133416	0.78827620	-4.12223921
H	2.06787815	2.30364114	-5.35049592
H	-6.59436827	3.61004527	-3.14271593
H	2.20140933	-3.18155773	5.96324169
H	-0.43504749	-1.80481886	4.88265220
H	-8.22684728	0.42246161	1.82118178
H	6.59436827	-3.61004527	3.14271593
H	8.96480151	-2.82671671	0.06937051
H	-2.20140933	3.18155773	-5.96324169
H	0.43504749	1.80481886	-4.88265220
H	-5.31133416	-0.78827620	4.12223921
H	-2.06787815	-2.30364114	5.35049592
H	-5.27324033	1.83122500	-2.09257964
H	-9.64373243	0.65186951	0.75090570
H	5.16993148	-4.28129895	3.95507491
H	7.25108301	-2.99334868	0.57218598
H	-3.86558530	2.73410584	-5.53768936
H	1.80098514	0.68896691	-4.65557480
H	1.50597103	-3.20513090	3.60576134
H	-0.61359156	1.22403636	-3.20037127
H	-1.07813696	-1.74739476	2.47393913
H	-5.02280479	1.75757977	-3.81374356
H	7.65174839	-2.76823811	-1.15522788
H	2.90162267	-4.24630435	3.79065971
H	5.28967055	-4.36648875	2.18511944
H	-2.54278987	1.57523564	-5.27812173
H	-4.07846399	-2.52478840	4.59284250
H	5.18792195	-1.88423720	-3.91791179
H	-9.19367067	-1.71254037	1.00913413
H	-1.41182755	-3.34859440	3.15457205
H	5.80681208	-3.27947178	-2.40165954
H	-5.99636168	-2.41123317	2.80986450
H	-8.90023554	-1.22007991	-0.68645278
H	-3.55206765	-3.99383570	3.79578314
H	0.89657921	-2.23958076	-2.25413108
H	1.77855565	-1.54441031	-4.52240051
H	2.75293944	-5.26096896	1.81121808
H	-0.61503286	-1.46291781	-3.02389114
H	5.10906455	-4.55313115	-1.37915813
H	-6.08514206	-3.71660346	4.01785865
H	3.91357747	-3.62897081	-4.06006428
H	-7.58076615	-3.59156389	0.79602026

H	-5.51957917	-4.05549238	2.36463451
H	5.72534609	-4.95065198	-3.00618768
H	-5.36351236	-1.89632089	-2.13602838
H	0.33781288	-2.57918246	-4.66036951
H	-5.96805503	-3.16230459	0.13902981
H	1.12434982	-3.93649431	-2.69536242
H	2.60971764	-5.45706926	-0.90757581
H	1.94511120	-3.25280229	-4.98210526
H	-7.40761909	-3.12476076	-0.93163078
H	-3.35986887	-5.00148491	1.73568298
H	3.24101971	-4.85969230	-3.00562147
H	-4.84035744	-2.00417335	-3.79538840
H	-2.25972786	-2.23253312	-4.95839017
H	-1.45174548	-3.48859565	-2.88344366
H	-3.39124125	-5.24009207	-0.97835729
H	-5.44524908	-4.44808626	-2.01244766
H	-6.54898238	-3.75766745	-3.22888041
H	-3.55155557	-3.43724437	-5.14245713
H	-2.82758555	-4.56306653	-3.02883781
H	-1.85305820	-3.93733605	-5.26187489
H	-5.03043815	-4.51389273	-3.73701659
N	3.18435707	2.10989387	-0.11908843
N	3.54647599	0.06225748	2.34605972
N	-3.03658116	2.36291079	-0.20884428
N	0.05401315	1.22915135	-0.07770066
N	-2.92031138	0.29286327	2.08125725
N	2.92031138	-0.29286327	-2.08125725
N	-0.05401315	-1.22915135	0.07770066
N	3.03658116	-2.36291079	0.20884428
N	-3.54647599	-0.06225748	-2.34605972
N	-3.18435707	-2.10989387	0.11908843
O	7.20834533	1.58059444	-0.44005014
O	7.80384277	-1.10301233	0.08695396
O	-7.80384277	1.10301233	-0.08695396
O	-7.20834533	-1.58059444	0.44005014
K	0.25097414	3.86669554	-0.30987416
K	5.17946095	-0.19124731	-0.14243241
K	-5.17946095	0.19124731	0.14243241
K	-0.25097414	-3.86669554	0.30987416
U	1.63149704	-0.06570547	0.19008348
U	-1.63149704	0.06570547	-0.19008348

Optimal geometries for II with PBE0/ECP60MDF

C	-6.34602089	-0.44223745	-4.94511626
C	-4.01688943	-4.49706475	-4.26233011
C	-4.20740667	0.65373837	-4.25440797
C	-5.40642155	-0.15543394	-3.77936345
C	-0.26415735	-0.64585298	-5.32004744
C	-3.16554908	-4.10945697	-3.05777016
C	-7.40809587	-3.05290746	-2.21550741
C	-6.54836324	-4.18141265	-0.15652534
C	-6.12886195	0.60659691	-2.67393740
C	-2.08064589	-3.11787452	-3.46103847
C	-0.33902149	1.86266745	-5.27505795
C	-2.55095027	-5.33896446	-2.40275500
C	-4.80955575	4.61619178	-3.40967554
C	-6.88758369	-2.84092133	-0.79780055
C	0.05635217	0.60317679	-4.51164436
C	4.32393598	-0.09704918	-5.62962380
C	3.94480834	2.38740672	-5.70536055
C	2.88781957	-5.35227930	-2.99816098
C	-0.64855202	0.55587201	-3.15525535
C	-7.93159574	-2.10337402	0.03618239
C	-3.91932299	4.55624449	-2.17501554
C	4.54041462	1.23054149	-4.91042050
C	2.81075179	-2.84969816	-3.00788018
C	-2.56016304	3.96183257	-2.52200218
C	-7.52559825	2.89775544	-0.18139939
C	1.08762824	-4.15960547	-1.73430977
C	2.53797598	-4.10830701	-2.18894556
C	-3.76535673	5.94489940	-1.56259238
C	6.02675439	1.47032052	-4.66489173
C	2.27228848	5.21483287	-3.21303111
C	-6.67179712	3.02974989	1.07525077
C	-3.50162310	-3.76741847	1.58484091
C	2.18416675	3.98729781	-2.31402070
C	6.47780052	-4.49645561	-1.44340902
C	7.31770303	-2.28524084	-2.24076902
C	-7.31770303	2.28524084	2.24076902
C	-6.47780052	4.49645561	1.44340902
C	-1.04606491	-4.13197746	1.31611108
C	1.04606491	4.13197746	-1.31611108
C	6.67179712	-3.02974989	-1.07525077
C	-2.18416675	-3.98729781	2.31402070
C	3.50162310	3.76741847	-1.58484091

C	-2.27228848	-5.21483287	3.21303111
C	-6.02675439	-1.47032052	4.66489173
C	3.76535673	-5.94489940	1.56259238
C	7.52559825	-2.89775544	0.18139939
C	-2.53797598	4.10830701	2.18894556
C	-1.08762824	4.15960547	1.73430977
C	-4.54041462	-1.23054149	4.91042050
C	2.56016304	-3.96183257	2.52200218
C	3.91932299	-4.55624449	2.17501554
C	7.93159574	2.10337402	-0.03618239
C	-2.81075179	2.84969816	3.00788018
C	-2.88781957	5.35227930	2.99816098
C	-3.94480834	-2.38740672	5.70536055
C	0.64855202	-0.55587201	3.15525535
C	-4.32393598	0.09704918	5.62962380
C	6.88758369	2.84092133	0.79780055
C	4.80955575	-4.61619178	3.40967554
C	-0.05635217	-0.60317679	4.51164436
C	2.55095027	5.33896446	2.40275500
C	6.54836324	4.18141265	0.15652534
C	7.40809587	3.05290746	2.21550741
C	0.33902149	-1.86266745	5.27505795
C	6.12886195	-0.60659691	2.67393740
C	2.08064589	3.11787452	3.46103847
C	3.16554908	4.10945697	3.05777016
C	0.26415735	0.64585298	5.32004744
C	5.40642155	0.15543394	3.77936345
C	4.20740667	-0.65373837	4.25440797
C	4.01688943	4.49706475	4.26233011
C	6.34602089	0.44223745	4.94511626
H	-5.81616423	-1.00797528	-5.72574139
H	-6.72486365	0.49243073	-5.38512460
H	-7.20355834	-1.04416656	-4.61190490
H	-3.40431831	-4.97366376	-5.04314594
H	-4.49779234	-3.60264440	-4.68445031
H	-4.80276738	-5.20549825	-3.95895190
H	-3.60476562	0.06502118	-4.96111346
H	-4.52488238	1.58052847	-4.75465478
H	-6.65039407	-3.54192570	-2.84346802
H	0.28712711	-0.62399253	-6.27162688
H	-1.33952728	-0.71392996	-5.53964144
H	-5.74432699	-4.67273397	-0.72122318
H	0.16711422	1.88692942	-6.25196572
H	-6.50354246	1.56834060	-3.05275763

H	-8.30385934	-3.69115286	-2.19380624
H	-1.39325354	-3.57668858	-4.18713655
H	-1.42630349	1.88931289	-5.44655504
H	-7.43099168	-4.83889989	-0.14000679
H	-2.52623671	-2.23216037	-3.93728655
H	-1.89154642	-5.87351752	-3.10303564
H	-3.58649140	0.92910340	-3.39228030
H	-6.98069687	0.03074998	-2.28656991
H	0.03729586	-1.54430668	-4.76378020
H	-3.34293856	-6.02806368	-2.07285892
H	-4.94521574	3.60936644	-3.82930846
H	-4.36779251	5.26227660	-4.18305543
H	-7.67614225	-2.09725025	-2.68548448
H	4.81452096	-0.09648817	-6.61508852
H	-6.20597519	-4.03688010	0.87781695
H	-5.79972259	5.01603849	-3.14466946
H	3.24918362	-0.28171698	-5.76310617
H	2.25015038	-5.42803135	-3.89170493
H	-0.04394208	2.75641683	-4.70813356
H	-5.44394434	0.83634030	-1.84406076
H	4.42847541	2.46417438	-6.69051411
H	-1.49844573	-2.79163155	-2.58652173
H	2.86656851	2.23914220	-5.86064105
H	-1.73121732	0.46181559	-3.30680024
H	-1.95985907	-5.04141984	-1.52539349
H	2.21322751	-2.85529390	-3.93136524
H	-8.87877714	-2.66176964	0.06749041
H	4.74151903	-0.92305338	-5.03352729
H	0.41604105	-4.23897698	-2.60063290
H	3.93857227	-5.32456264	-3.32305920
H	-2.67740298	2.96463711	-2.96745673
H	2.74146736	-6.25486651	-2.38666423
H	-7.02549660	3.39077995	-1.02604736
H	-2.02258612	4.59902947	-3.24012458
H	-7.66710057	1.83691307	-0.43856615
H	4.08564468	3.33817406	-5.17168390
H	-0.28032779	-0.31591013	-2.58535413
H	-8.51697262	3.35244040	-0.03267670
H	3.87310117	-2.79278721	-3.29121925
H	-8.13358375	-1.10713017	-0.38701914
H	6.57649187	1.55818239	-5.61364111
H	-0.44320189	1.47980937	-2.58608339
H	-7.58752543	-1.98538369	1.07703072
H	-3.28440111	6.63219688	-2.27534972

H	1.33728954	5.33449075	-3.78014873
H	-4.75285636	6.35441745	-1.30078018
H	2.54221339	-1.93870380	-2.45359279
H	0.92743187	-5.04172222	-1.09608004
H	3.09866912	5.11200599	-3.93226264
H	-3.75302142	-4.64646419	0.97614270
H	0.80954571	-3.25419734	-1.17187473
H	-1.93655856	3.86282387	-1.62144610
H	5.85812396	-4.59403640	-2.34523433
H	6.66003435	-2.30734218	-3.12341866
H	6.46702214	0.63066160	-4.10264244
H	-3.43083824	-2.91480141	0.89450056
H	6.17658151	2.39862897	-4.09188483
H	-3.16094556	5.89620716	-0.64678265
H	-1.23157151	-4.97681825	0.63603570
H	0.10439255	4.32411303	-1.84663923
H	-7.52127255	1.23475640	1.97460149
H	2.43873441	6.12500541	-2.61747011
H	7.45246448	-4.96976383	-1.63394920
H	-5.98052653	5.04324505	0.63006698
H	8.28012680	-2.74138810	-2.51569289
H	-4.31900531	-3.59337000	2.30127044
H	-0.92156274	-3.21819250	0.71445520
H	-8.28012680	2.74138810	2.51569289
H	-7.45246448	4.96976383	1.63394920
H	5.98052653	-5.04324505	-0.63006698
H	7.52127255	-1.23475640	-1.97460149
H	-2.43873441	-6.12500541	2.61747011
H	4.31900531	3.59337000	-2.30127044
H	3.16094556	-5.89620716	0.64678265
H	0.92156274	3.21819250	-0.71445520
H	-6.17658151	-2.39862897	4.09188483
H	1.23157151	4.97681825	-0.63603570
H	-6.46702214	-0.63066160	4.10264244
H	-6.66003435	2.30734218	3.12341866
H	-0.10439255	-4.32411303	1.84663923
H	-5.85812396	4.59403640	2.34523433
H	3.43083824	2.91480141	-0.89450056
H	-3.09866912	-5.11200599	3.93226264
H	3.75302142	4.64646419	-0.97614270
H	4.75285636	-6.35441745	1.30078018
H	1.93655856	-3.86282387	1.62144610
H	-0.80954571	3.25419734	1.17187473
H	3.28440111	-6.63219688	2.27534972

H	-6.57649187	-1.55818239	5.61364111
H	-1.33728954	-5.33449075	3.78014873
H	-0.92743187	5.04172222	1.09608004
H	8.51697262	-3.35244040	0.03267670
H	7.58752543	1.98538369	-1.07703072
H	8.13358375	1.10713017	0.38701914
H	7.66710057	-1.83691307	0.43856615
H	-2.54221339	1.93870380	2.45359279
H	-4.08564468	-3.33817406	5.17168390
H	7.02549660	-3.39077995	1.02604736
H	-3.87310117	2.79278721	3.29121925
H	-2.74146736	6.25486651	2.38666423
H	0.44320189	-1.47980937	2.58608339
H	8.87877714	2.66176964	-0.06749041
H	2.02258612	-4.59902947	3.24012458
H	-3.93857227	5.32456264	3.32305920
H	0.28032779	0.31591013	2.58535413
H	-4.74151903	0.92305338	5.03352729
H	2.67740298	-2.96463711	2.96745673
H	-0.41604105	4.23897698	2.60063290
H	-4.42847541	-2.46417438	6.69051411
H	1.95985907	5.04141984	1.52539349
H	-2.86656851	-2.23914220	5.86064105
H	-2.21322751	2.85529390	3.93136524
H	5.79972259	-5.01603849	3.14466946
H	1.73121732	-0.46181559	3.30680024
H	-2.25015038	5.42803135	3.89170493
H	-4.81452096	0.09648817	6.61508852
H	-3.24918362	0.28171698	5.76310617
H	6.20597519	4.03688010	-0.87781695
H	1.49844573	2.79163155	2.58652173
H	5.44394434	-0.83634030	1.84406076
H	7.67614225	2.09725025	2.68548448
H	0.04394208	-2.75641683	4.70813356
H	4.36779251	-5.26227660	4.18305543
H	4.94521574	-3.60936644	3.82930846
H	3.34293856	6.02806368	2.07285892
H	6.98069687	-0.03074998	2.28656991
H	8.30385934	3.69115286	2.19380624
H	7.43099168	4.83889989	0.14000679
H	-0.03729586	1.54430668	4.76378020
H	1.89154642	5.87351752	3.10303564
H	6.50354246	-1.56834060	3.05275763
H	3.58649140	-0.92910340	3.39228030

H	1.42630349	-1.88931289	5.44655504
H	2.52623671	2.23216037	3.93728655
H	-0.16711422	-1.88692942	6.25196572
H	5.74432699	4.67273397	0.72122318
H	1.39325354	3.57668858	4.18713655
H	6.65039407	3.54192570	2.84346802
H	-0.28712711	0.62399253	6.27162688
H	1.33952728	0.71392996	5.53964144
H	4.52488238	-1.58052847	4.75465478
H	4.80276738	5.20549825	3.95895190
H	3.60476562	-0.06502118	4.96111346
H	4.49779234	3.60264440	4.68445031
H	7.20355834	1.04416656	4.61190490
H	3.40431831	4.97366376	5.04314594
H	6.72486365	-0.49243073	5.38512460
H	5.81616423	1.00797528	5.72574139
N	-0.04891013	-1.18382159	-0.35905239
N	0.04891013	1.18382159	0.35905239
O	-4.94460430	-1.42874902	-3.33090378
O	-4.03342029	-3.54027239	-2.07237041
O	-5.72109897	-2.00474442	-0.78070901
O	1.47348596	0.58616029	-4.33856527
O	-3.31469961	-1.04744851	-1.17081158
O	-4.60380568	3.70326751	-1.25323020
O	1.88026038	2.89148601	-3.18599381
O	3.94773314	1.14491661	-3.61009163
O	-3.09459828	1.69994214	-0.13029029
O	3.36923753	-4.16435529	-1.02243642
O	-5.41653116	2.37587774	0.84549611
O	2.28884649	0.62024265	-1.71987411
O	5.41653116	-2.37587774	-0.84549611
O	-2.28884649	-0.62024265	1.71987411
O	-3.36923753	4.16435529	1.02243642
O	3.09459828	-1.69994214	0.13029029
O	-3.94773314	-1.14491661	3.61009163
O	-1.88026038	-2.89148601	3.18599381
O	4.60380568	-3.70326751	1.25323020
O	3.31469961	1.04744851	1.17081158
O	5.72109897	2.00474442	0.78070901
O	-1.47348596	-0.58616029	4.33856527
O	4.03342029	3.54027239	2.07237041
O	4.94460430	1.42874902	3.33090378
Si	-4.43814244	-1.96484212	-1.85517533
Si	2.35091452	1.32097447	-3.15987434

Si	-4.03874780	2.97851600	0.11282556
Si	4.03874780	-2.97851600	-0.11282556
Si	-2.35091452	-1.32097447	3.15987434
Si	4.43814244	1.96484212	1.85517533
K	-5.01430502	-0.27293858	1.21062445
K	5.01430502	0.27293858	-1.21062445
U	-1.60694058	0.07417576	-0.23643750
U	1.60694058	-0.07417576	0.23643750

Optimal geometries for III with PBE0/ECP60MDF

C	2.27907454	-3.49367048	-5.63079101
C	6.66233183	-0.99589291	-2.68494810
C	2.00323688	-3.55813168	-4.13064479
C	-1.01614276	-5.11930486	-4.85217064
C	3.13989192	-2.90872198	-3.35500493
C	7.68935972	2.65785069	-2.69958609
C	-0.94777529	-3.59576329	-4.92806894
C	6.99071187	-0.30484360	-1.36456613
C	7.03676797	-1.30914358	-0.22010131
C	1.54038386	0.54637111	-4.68377514
C	-2.33268355	-2.96438333	-4.95327506
C	6.20971092	2.53618384	-2.34595416
C	0.30529350	-0.34673984	-4.72408713
C	0.42518888	-4.85507071	-1.47553816
C	3.51295370	0.59044508	-2.40153323
C	2.77210716	1.80995563	-2.97946408
C	5.61245819	3.89717573	-1.99709088
C	3.49931183	-3.32020312	0.15568745
C	-0.11718580	-3.46981295	-1.80665208
C	0.73804311	2.71898806	-3.89505268
C	7.61591966	2.25002097	0.93549219
C	-2.71430380	2.57753497	-5.73699164
C	2.74585019	-5.26840635	1.53706313
C	3.51301493	-3.94959550	1.54120197
C	6.17833288	1.75676700	0.79263134
C	-1.60737425	-3.38459635	-1.49441291
C	-3.40894423	-0.07902012	-3.48267899
C	-2.89927776	3.54943051	-4.57407090
C	5.36535189	-1.44952995	3.14077224
C	-0.10617565	3.19124174	-2.71963404
C	5.19045339	2.76159678	1.37162598
C	4.14303368	-4.41192550	4.78142126
C	-4.14303368	4.41192550	-4.78142126

C	3.93331358	-1.12729607	2.71879896
C	-3.93331358	1.12729607	-2.71879896
C	-5.19045339	-2.76159678	-1.37162598
C	-5.36535189	1.44952995	-3.14077224
C	2.89927776	-3.54943051	4.57407090
C	0.10617565	-3.19124174	2.71963404
C	3.40894423	0.07902012	3.48267899
C	-6.17833288	-1.75676700	-0.79263134
C	-7.61591966	-2.25002097	-0.93549219
C	1.60737425	3.38459635	1.49441291
C	-3.51301493	3.94959550	-1.54120197
C	2.71430380	-2.57753497	5.73699164
C	-2.74585019	5.26840635	-1.53706313
C	-0.73804311	-2.71898806	3.89505268
C	-5.61245819	-3.89717573	1.99709088
C	-3.49931183	3.32020312	-0.15568745
C	0.11718580	3.46981295	1.80665208
C	-2.77210716	-1.80995563	2.97946408
C	-0.42518888	4.85507071	1.47553816
C	-3.51295370	-0.59044508	2.40153323
C	-6.20971092	-2.53618384	2.34595416
C	2.33268355	2.96438333	4.95327506
C	-0.30529350	0.34673984	4.72408713
C	-7.03676797	1.30914358	0.22010131
C	-1.54038386	-0.54637111	4.68377514
C	-7.68935972	-2.65785069	2.69958609
C	-6.99071187	0.30484360	1.36456613
C	0.94777529	3.59576329	4.92806894
C	1.01614276	5.11930486	4.85217064
C	-3.13989192	2.90872198	3.35500493
C	-2.00323688	3.55813168	4.13064479
C	-6.66233183	0.99589291	2.68494810
C	-2.27907454	3.49367048	5.63079101
H	3.27309788	-3.91170060	-5.86721735
H	1.54094998	-4.05673635	-6.22080104
H	7.39857021	-1.78340968	-2.92289129
H	2.27489380	-2.45464113	-6.00096817
H	6.64693600	-0.29393407	-3.53452159
H	4.08670927	-3.46401034	-3.46772807
H	-0.01898869	-5.58683305	-4.87223986
H	5.67462805	-1.48361287	-2.64245072
H	-1.59162053	-5.53628720	-5.69667606
H	1.96121815	-4.62605559	-3.84586482
H	8.10817113	1.70790490	-3.06384196

H	-0.46709944	-3.33900302	-5.89059243
H	7.84720001	3.41030250	-3.49213017
H	7.68328212	-2.17029987	-0.46388744
H	3.32076911	-1.88553980	-3.71332238
H	7.99677635	0.14420276	-1.45694989
H	0.37386034	-0.97215672	-5.63566589
H	1.68310553	1.09477076	-5.63617036
H	2.41773300	-0.09822312	-4.54528739
H	-1.51645379	-5.45589454	-3.92933175
H	-2.93376977	-3.36123416	-5.78998805
H	8.29473273	2.97116968	-1.83427367
H	5.69058073	2.16929066	-3.25287671
H	2.92385939	-2.84218269	-2.27984839
H	4.23251069	0.22631871	-3.15848062
H	6.03595816	-1.71374501	0.00123207
H	-0.00928252	-5.62746968	-2.12913741
H	1.51768646	-4.91058897	-1.58256365
H	3.39834047	2.34517767	-3.72173761
H	3.81505877	-4.02961149	-0.62609487
H	-2.27212077	-1.87471611	-5.07348186
H	7.41873303	-0.86587560	0.71146181
H	-0.58244170	0.29691231	-4.89120339
H	2.73515248	-0.21446490	-2.38299466
H	8.34533038	1.52845992	0.53577696
H	5.67850057	4.59936128	-2.84637686
H	3.18439304	-5.98953143	0.82561243
H	-2.89988404	-3.16079742	-4.03028337
H	-2.56554663	3.11527130	-6.68908793
H	0.05359310	2.50498100	-4.72640414
H	-1.85139632	1.90891138	-5.59393963
H	0.18509682	-5.13604128	-0.43591409
H	4.19331699	-2.46433118	0.08845332
H	1.44943730	3.49624954	-4.23938698
H	2.47561838	-2.98631828	-0.09629709
H	4.56216124	-4.15534567	1.82084688
H	7.76980630	3.20048664	0.39951960
H	6.14872853	4.36371600	-1.15465883
H	-3.60150453	1.93737148	-5.86756327
H	-2.18255608	-4.16373258	-2.01745045
H	0.39772490	-2.73831755	-1.14869244
H	4.55134323	3.82452334	-1.70986490
H	2.60137515	2.53192226	-2.16388313
H	-3.37052503	0.11405167	-4.56434634
H	6.08135326	0.83178577	1.38977217

H	1.70038423	-5.11351231	1.23044693
H	2.73216750	-5.74929099	2.52728300
H	5.78798851	-2.30221310	2.58868034
H	7.87499917	2.43067009	1.99300632
H	-2.38938829	-0.38787928	-3.19431163
H	-2.02481916	-2.41719320	-1.81938405
H	4.26512478	-5.17238102	3.99640820
H	-4.05994247	-0.95592671	-3.33662951
H	-2.02760229	4.23229076	-4.57209938
H	-1.79547102	-3.53317613	-0.41162183
H	6.03187699	-0.58690396	2.97406483
H	-4.09583672	4.94286170	-5.74798487
H	3.96505616	-0.84733566	1.64646762
H	5.13066989	3.68915660	0.78244385
H	-0.46108157	4.21551758	-2.93830518
H	5.06248374	-3.80660707	4.79330835
H	0.56219484	3.32889574	-1.83863177
H	-5.46493294	-3.04628183	-2.40271720
H	5.41675701	-1.68969026	4.21416721
H	4.17601165	2.34332854	1.44114067
H	-5.06248374	3.80660707	-4.79330835
H	-5.41675701	1.68969026	-4.21416721
H	4.09583672	-4.94286170	5.74798487
H	5.46493294	3.04628183	2.40271720
H	-4.17601165	-2.34332854	-1.44114067
H	-5.13066989	-3.68915660	-0.78244385
H	0.46108157	-4.21551758	2.93830518
H	-0.56219484	-3.32889574	1.83863177
H	-6.03187699	0.58690396	-2.97406483
H	-4.26512478	5.17238102	-3.99640820
H	2.02760229	-4.23229076	4.57209938
H	-3.96505616	0.84733566	-1.64646762
H	-7.87499917	-2.43067009	-1.99300632
H	4.05994247	0.95592671	3.33662951
H	1.79547102	3.53317613	0.41162183
H	-5.78798851	2.30221310	-2.58868034
H	-2.73216750	5.74929099	-2.52728300
H	2.38938829	0.38787928	3.19431163
H	2.02481916	2.41719320	1.81938405
H	-6.08135326	-0.83178577	-1.38977217
H	-7.76980630	-3.20048664	-0.39951960
H	-1.70038423	5.11351231	-1.23044693
H	3.37052503	-0.11405167	4.56434634
H	-6.14872853	-4.36371600	1.15465883

H	3.60150453	-1.93737148	5.86756327
H	-4.55134323	-3.82452334	1.70986490
H	-4.56216124	4.15534567	-1.82084688
H	2.18255608	4.16373258	2.01745045
H	-2.60137515	-2.53192226	2.16388313
H	2.56554663	-3.11527130	6.68908793
H	-0.39772490	2.73831755	1.14869244
H	1.85139632	-1.90891138	5.59393963
H	-8.34533038	-1.52845992	-0.53577696
H	-1.44943730	-3.49624954	4.23938698
H	-2.47561838	2.98631828	0.09629709
H	-0.18509682	5.13604128	0.43591409
H	-5.67850057	-4.59936128	2.84637686
H	-4.19331699	2.46433118	-0.08845332
H	-0.05359310	-2.50498100	4.72640414
H	-3.18439304	5.98953143	-0.82561243
H	2.89988404	3.16079742	4.03028337
H	-7.41873303	0.86587560	-0.71146181
H	0.58244170	-0.29691231	4.89120339
H	-2.73515248	0.21446490	2.38299466
H	2.27212077	1.87471611	5.07348186
H	-3.81505877	4.02961149	0.62609487
H	-8.29473273	-2.97116968	1.83427367
H	-3.39834047	-2.34517767	3.72173761
H	0.00928252	5.62746968	2.12913741
H	-1.51768646	4.91058897	1.58256365
H	-6.03595816	1.71374501	-0.00123207
H	-5.69058073	-2.16929066	3.25287671
H	2.93376977	3.36123416	5.78998805
H	1.51645379	5.45589454	3.92933175
H	-4.23251069	-0.22631871	3.15848062
H	-2.92385939	2.84218269	2.27984839
H	-7.84720001	-3.41030250	3.49213017
H	-7.99677635	-0.14420276	1.45694989
H	-1.68310553	-1.09477076	5.63617036
H	-2.41773300	0.09822312	4.54528739
H	-0.37386034	0.97215672	5.63566589
H	-7.68328212	2.17029987	0.46388744
H	-8.10817113	-1.70790490	3.06384196
H	-3.32076911	1.88553980	3.71332238
H	1.59162053	5.53628720	5.69667606
H	0.46709944	3.33900302	5.89059243
H	-5.67462805	1.48361287	2.64245072
H	0.01898869	5.58683305	4.87223986

H	-1.96121815	4.62605559	3.84586482
H	-6.64693600	0.29393407	3.53452159
H	-4.08670927	3.46401034	3.46772807
H	-7.39857021	1.78340968	2.92289129
H	-2.27489380	2.45464113	6.00096817
H	-1.54094998	4.05673635	6.22080104
H	-3.27309788	3.91170060	5.86721735
Li	2.97222100	-0.67557154	-0.47574241
Li	2.70585748	1.58302384	-0.00243449
Li	-2.70585748	-1.58302384	0.00243449
Li	-2.97222100	0.67557154	0.47574241
N	0.16771362	-1.11965378	-3.49615344
N	1.46426257	1.48513462	-3.55369256
N	4.09934281	0.78437751	-1.08661021
N	-1.17293760	2.24733059	-2.42648891
N	1.31763925	0.26117949	-0.04339479
N	1.17293760	-2.24733059	2.42648891
N	-1.31763925	-0.26117949	0.04339479
N	-4.09934281	-0.78437751	1.08661021
N	-1.46426257	-1.48513462	3.55369256
N	-0.16771362	1.11965378	3.49615344
Si	0.26735642	-2.85105170	-3.60760626
Si	5.78614438	1.18070716	-1.01256670
Si	2.80230005	-2.69179721	2.83290091
Si	-2.80230005	2.69179721	-2.83290091
Si	-5.78614438	-1.18070716	1.01256670
Si	-0.26735642	2.85105170	3.60760626
U	-0.14801286	0.31085015	-1.65522429
U	0.14801286	-0.31085015	1.65522429
